# Prion-Associated Toxicity is Rescued by Elimination of Cotranslational Chaperones

**DOI:** 10.1371/journal.pgen.1006431

**Published:** 2016-11-09

**Authors:** Kathryn M. Keefer, Heather L. True

**Affiliations:** Department of Cell Biology and Physiology, Washington University School of Medicine, St. Louis, Missouri, United States of America; Northwestern University, UNITED STATES

## Abstract

The nascent polypeptide-associated complex (NAC) is a highly conserved but poorly characterized triad of proteins that bind near the ribosome exit tunnel. The NAC is the first cotranslational factor to bind to polypeptides and assist with their proper folding. Surprisingly, we found that deletion of NAC subunits in *Saccharomyces cerevisiae* rescues toxicity associated with the strong [*PSI*+] prion. This counterintuitive finding can be explained by changes in chaperone balance and distribution whereby the folding of the prion protein is improved and the prion is rendered nontoxic. In particular, the ribosome-associated Hsp70 Ssb is redistributed away from Sup35 prion aggregates to the nascent chains, leading to an array of aggregation phenotypes that can mimic both overexpression and deletion of Ssb. This toxicity rescue demonstrates that chaperone modification can block key steps of the prion life cycle and has exciting implications for potential treatment of many human protein conformational disorders.

## Author Summary

Misfolded proteins can be toxic to cells, causing pathologies such as Alzheimer’s disease, Parkinson’s disease, prion diseases, and ALS. One mechanism by which organisms combat protein misfolding involves molecular chaperones, proteins that help other proteins fold correctly. Here, we describe a novel role for a family of chaperones called the nascent polypeptide-associated complex (NAC). The NAC is a group of proteins that exist in all multicellular organisms, yet we do not fully understand its function. Using yeast as a model system, we have found that deletion of NAC subunits can reduce the toxicity associated with misfolded proteins. This work has implications for human protein misfolding diseases, as modulation of the NAC may present a viable therapeutic avenue by which to slow the progression of neurodegeneration and other protein conformational disorders.

## Introduction

Protein synthesis is an essential process undertaken by all organisms, but its necessity also presents cells with a myriad of challenges. An extensive network of molecular machines is active throughout translation, folding, and degradation in order to preserve protein homeostasis (proteostasis). Perturbations to that machinery can have ripple effects that impact many cellular systems.

Misfolded proteins are one such challenge to proteostasis. Improperly folded proteins are generally non-functional; thus, the correct folding and trafficking of polypeptides is essential to the maintenance of cellular viability [1]. Protein misfolding can lead to the induction of cellular stress responses, apoptosis, and cell death. In humans, protein misfolding diseases include Alzheimer’s disease, Parkinson’s disease, Huntington’s disease, and prion diseases such as Creutzfeldt-Jakob disease [1–3]. The complexity of protein folding is mirrored by the complexity of these incurable diseases; thus, increased understanding of the molecular basis of folding and misfolding will be crucial to improved treatment of various pathologies.

Prions are a subset of misfolded proteins that are self-templating and stably propagated from cell to cell. In yeast, the intrinsically disordered domain of the translation termination factor Sup35 misfolds and aggregates to form the [*PSI*+] prion, which is cytoplasmically inherited via amyloid seeds [4–6]. [*PSI*+] is toxic under certain circumstances, including Sup35 overexpression, due to severe disruption of proteostasis as a consequence of excessive aggregation of Sup35 [7,8].

Nascent polypeptides begin to fold cotranslationally before protein synthesis has been completed by the ribosome [9]. Sup35, not unlike other proteins, faces folding challenges immediately upon emergence from the ribosome exit tunnel. Proteins are protected from early misfolding by ribosome-bound protein chaperone families [10]. First, the nascent polypeptide-associated complex (NAC) interacts with nascent chains [11], followed by the ribosome-associated complex (RAC) and the Hsp70 Ssb [12–15]. Cotranslational chaperone factors are of keen interest in the area of protein aggregation, as they have a bias towards substrates that are intrinsically disordered and amyloidogenic [16]. The NAC and Ssb-RAC systems are components of a larger molecular chaperone network within *Saccharomyces cerevisiae* [10,17]. However, the interactions between cotranslational folding factors and other players in the chaperone network have yet to be fully elucidated.

Here, we describe a surprising mechanism for preventing aggregation-related cytotoxicity by manipulating cotranslational folding pathways. We utilized the [*PSI*+] prion as a model for protein misfolding and a reporter for the activities of the chaperone network. We screened for factors that, when disrupted, rescued the prion-dependent toxicity and restored viability. Surprisingly, disruption of β-NAC, a subunit of the NAC chaperone complex, was identified as one of the rescuing mutants in our screen. This counterintuitive result suggests that depletion of chaperones can, in some cases, rescue defects associated with misfolded proteins.

Indeed, we found that deletion of NAC subunits has significant impact on the localization and activity of other cytosolic chaperones, the Hsp70 family in particular. We propose that altered localization and activity of chaperones can aid cells in the ability to maintain proteostasis when faced with severe folding challenges. As such, inhibition of the NAC presents a novel avenue for investigation into therapeutics to treat protein conformational disorders that may slow further aggregation of amyloidogenic proteins and suspend disease progression.

## Results

### Disruption of NAC subunits rescues toxicity associated with the [*PSI*+] prion

**Fig 1:**
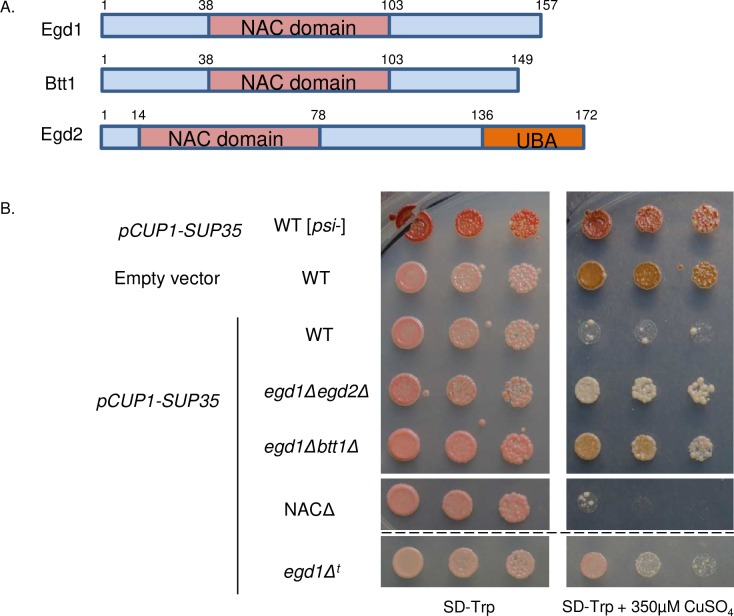
Disruption of NAC subunits rescues toxicity associated with the [*PSI*+] prion. (A) The nascent-polypeptide associated complex consists of the proteins Egd1 (β), Btt1 (β’), and Egd2 (α) that share homology within their NAC domains. (B) Sup35 under control of the copper-inducible promoter CUP1 was induced in [*psi*-] WT, [*PSI*+] WT, and [*PSI*+] NAC deletion strains by growth on selective media containing 350μM CuSO4. The empty vector (EV) control contained the pCUP1 vector. Single horizontal white line separates non-contiguous spots from the same plates. Dashed black line separates spots from different plate of identical media. The “*egd1Δ*^t^” designation indicates the strain isolated from the transposon screen.

We set out to identify factors that modulate the toxic misfolding environment associated with the [*PSI*+] prion. Though [*PSI*+] is generally well-tolerated by cells, the overexpression of Sup35 in [*PSI*+] cells is cytotoxic [7]. To identify factors that could rescue this toxicity, we overexpressed Sup35 from a copper-inducible promoter and screened for colonies that overcame the toxicity phenotype while retaining the “strong” variant of [*PSI*+] (S1A Fig). Upon sequencing, two toxicity-suppressing candidate colonies contained single gene disruptions of *EGD1*. The *EGD1* gene encodes Egd1, the β-NAC subunit (Fig 1A). The NAC is comprised of three subunits: Egd1 (β subunit), Egd2 (α subunit), and Btt1 (β’ subunit), which are together known to play an important role in cotranslational folding and protein homeostasis (proteostasis) [18]. Our results indicate, for the first time, that deletion of NAC subunits may help to improve cellular health in the face of misfolding stress.

Intrigued by this result, we tested whether deletion of other NAC subunits also rescued the [*PSI*+]-dependent toxicity caused by Sup35 overexpression [7,8]. We theorized that double- or triple-deletions, which would not be recovered by our screen, may exhibit stronger phenotypes. We created yeast strains containing combinatorial deletions of all NAC subunits, hereafter referred to as “NAC deletion strains,” and tested them for growth in the presence of toxic Sup35 aggregates. We found that two double deletions, *egd1Δegd2Δ* and *egd1Δbtt1Δ*, strongly rescued the toxicity caused by the overexpression of Sup35 in [*PSI*+] cells (Fig 1B). Interestingly, other deletion combinations and deletion of the whole NAC did not detectably overcome the toxicity, potentially due to individual subunit interactions that are not yet understood (S1B Fig).

### Nonsense suppression and prion status are not changed as a result of NAC subunit deletion

It has been previously shown that impaired translation termination is responsible for the toxicity phenotype in [*PSI*+] cells overexpressing Sup35 [7]. Therefore, we hypothesized that a decrease in stop codon readthrough may be responsible for the toxicity rescue in the NAC deletion strains.

**Fig 2:**
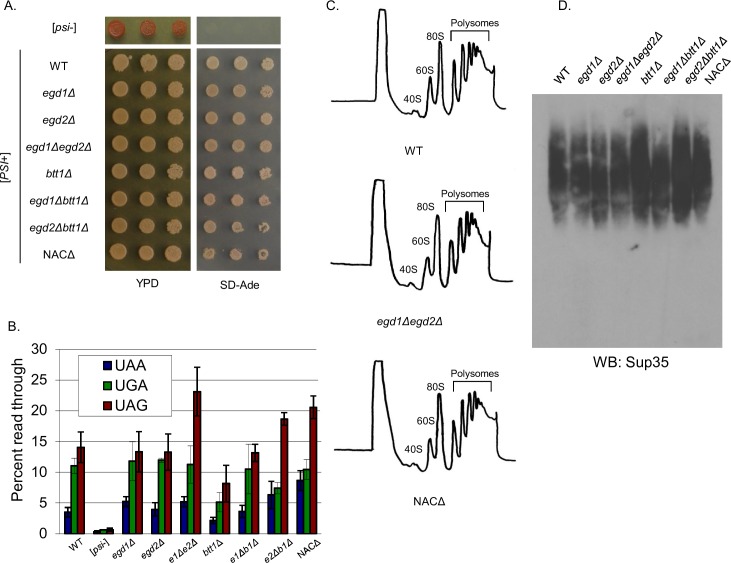
Nonsense suppression and prion status are not changed as a result of NAC subunit deletion. (A) [*PSI*+] NAC deletion strains were tested for growth on YPD and SD-Ade to monitor nonsense suppression of the *ade1-14* allele. (B) Stop codon readthrough is not significantly altered in NAC deletion strains relative to the WT. Expression of PGK(stop)LacZ fusion proteins was monitored by a β-galactosidase assay [50]. Data are represented as mean ± SEM. (C) Ribosome profiling revealed no differences in ribosome or polysome formation between the NAC deletion strains. (D) SDD-AGE shows that Sup35 aggregates in the NAC deletion strains are not changed relative to the WT, and all strains retain the “strong” strain of the [*PSI*+] prion.

To test this, we utilized a well-characterized genetic assay: the *ade1-14* allele, which contains a premature stop codon in the *ADE1* open reading frame. Yeast carrying the *ade1-14* allele are unable to complete adenine biosynthesis, resulting in accumulation of a red pigment in cells grown on rich media that have faithful translation termination. The nonsense suppression that occurs in *ade1-14* [*PSI*+] cells leads to completion of the adenine biosynthesis pathway, thus the colonies are white in color and are able to grow on media lacking adenine (SD-Ade) [19]. We spotted [*PSI*+] NAC deletion strains onto rich media (YPD) and SD-Ade plates to assess their nonsense suppression phenotypes. All NAC deletion strains formed white colonies and grew strongly on SD-Ade (Fig 2A), indicating similar levels of nonsense suppression in all strains in the context of endogenous Sup35.

To assess nonsense suppression quantitatively, we measured stop codon readthrough via the expression of β-galactosidase from a set of reporter plasmids [20]. Quantification indicated that there was no significant change in nonsense suppression in any of the NAC deletion strains relative to the WT [*PSI*+] control (Fig 2B). We concluded that a decrease in nonsense suppression was not the mechanism by which the NAC deletion strains rescued [*PSI*+]-associated toxicity.

We considered the possibility that a global change in translation was playing a role in toxicity rescue. The quintuple deletion of subunits in a *nacΔssbΔ* strain has been demonstrated to cause a defect in ribosome biogenesis [21], and fewer translating ribosomes may allow cells to tolerate reduced Sup35 function and withstand toxicity by improving the ratio of translation termination factors. We analyzed ribosome profiles for all NAC deletion strains and found no differences in the peak heights or integrated peak areas (Fig 2C), nor did we observe the formation of ribosomal half-mers. Thus, translation is not globally perturbed in the NAC deletion strains relative to WT.

We next questioned whether the toxicity rescue indicated a change in prion variant of the NAC deletion strains. To assess the Sup35 aggregates biochemically, we used semi-denaturing detergent agarose gel electrophoresis (SDD-AGE). We found that the overall distribution of SDS-sensitive population of Sup35 aggregates did not change following growth in media without copper (Fig 2D), confirming that all NAC deletion strains retained the strong [*PSI*+] prion variant. Thus, the toxicity rescue phenotype exhibited by the NAC deletion strains was not due to loss or weakening of the [*PSI*+] prion.

### Prion aggregates are visibly altered due to NAC subunit deletion

**Fig 3:**
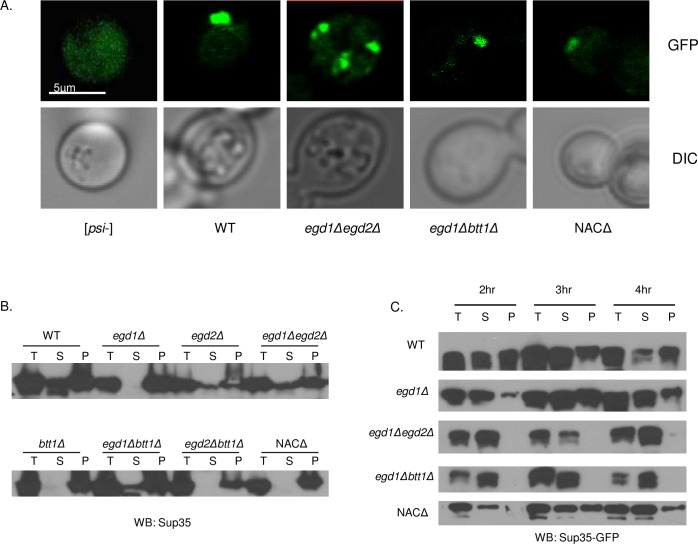
NAC deletion strains retain [*PSI*+] despite altered Sup35 solubility. (A) NAC deletion strains containing pCUP1-*SUP35-GFP* were grown in selective media in the presence of 50μM CuSO4. Two-dimensional images were taken with an Olympus FV1200 laser scanning microscope with a 100X oil immersion objective. The *egd1Δegd2Δ* strain showed a greater population of aggregates than the WT or other NAC deletion strains. (B) Solubility of Sup35 in [*PSI*+] lysates of indicated strains. Total (T), supernatant (S), and pellet (P) fractions were subjected to SDS-PAGE and Western blot. With endogenous levels of protein expression, all NAC deletion strains show insoluble Sup35. The *egd1Δegd2Δ* and *egd1Δbtt1Δ* strains exhibit more soluble Sup35 than the other NAC deletion strains. (C) NAC deletion strains were grown overnight in selective media; Sup35 overexpression was induced by the addition of CuSO_4_ to a final concentration of 50μM at time = 0. Solubility assays were performed on cells collected at indicated timepoints following Sup35 induction. The *egd1Δegd2Δ* and *egd1Δbtt1Δ* strains exhibited a defect in joining of nascent Sup35 to existing aggregates.

We were surprised that the NAC deletions rescued [*PSI*+]-related Sup35 overexpression toxicity without observable changes to the prion or to nonsense suppression. To further investigate the reduction of Sup35 toxicity, we examined the aggregation and solubility of the Sup35 prion aggregates in all NAC deletion strains. We sought to do so by a method that would allow observation of intact aggregates in cells, rather than simply the SDS-resistant aggregates detected by SDD-AGE (Fig 2D). We transformed a copper inducible, GFP-tagged Sup35 (pRS314CUP1 *Sup35GFP*) into the WT and NAC deletion strains. We induced Sup35-GFP expression by the addition of small amounts (V_f_ = 50μM) of CuSO_4_ to the culture media and monitored GFP localization by fluorescence microscopy. Sup35-GFP exhibited a diffuse pattern of fluorescence in [*psi*-] cells (Fig 3A), consistent with non-aggregated Sup35. By contrast, Sup35-GFP was observed in a single fluorescent focus in the WT [*PSI*+] strain (Fig 3A). Most NAC deletion cells also contained one major fluorescent puncta. However, the *egd1Δegd2Δ* strain harbored multiple fluorescent puncta throughout the cytoplasm (Fig 3A, S2A Fig). Interestingly, this phenotype was not apparent in the toxicity-rescuing *egd1Δbtt1Δ* deletion, suggesting that a similar change in aggregate distribution was not the rescuing effect. However, it is plausible that there is an altered aggregation pattern that is too subtle to be detected via fluorescence microscopy. Importantly, the WT and all NAC [*PSI*+] deletion strains exhibit insoluble Sup35 at steady-state levels of Sup35 expression (Fig 3B), as expected with the presence of the [*PSI*+] prion.

We then asked if the multiple aggregates in the *egd1Δegd2Δ* strain were due to altered joining of monomeric Sup35 to existing amyloid structures. To address this question, we induced the expression of Sup35-GFP and tracked its solubility over time. In lysates of WT [*PSI*+] strains, an abundance of Sup35-GFP appears in the pellet fraction at two hours post-induction of Sup35-GFP expression due to formation of insoluble aggregates (Fig 3C, S2B Fig). Remarkably, we did not detect Sup35-GFP in the pelleted fractions of *egd1Δegd2Δ* or *egd1Δbtt1Δ* strains up to four hours post-induction (Fig 3C), consistent with at least transiently enhanced solubility of Sup35. The amount of induced Sup35-GFP was consistent between all tested strains (S2C Fig). One interpretation of this result is that the joining of newly synthesized Sup35 to prion aggregates is delayed in the *egd1Δegd2Δ* or *egd1Δbtt1Δ* strains. We hypothesize that the structure of nascent Sup35 in the NAC deletion strains renders Sup35 impaired in joining to pre-existing aggregates due to altered, and possibly improved, folding of nascent Sup35. This is supported by the decrease in *de novo* [*PSI*+] induction in the toxicity-rescuing NAC deletion strains (S2D Fig).

### Chaperone balance is altered in NAC deletion strains

We next considered the possibility that the putative changes to Sup35 structure were brought about by altered interactions with chaperone proteins. Sup35 is a client of many cytosolic chaperones [22–24]. We reasoned that NAC deletion may spur a compensatory response of other molecular chaperones such that the deletion strains may, in fact, be folding some proteins more efficiently than WT.

When the NAC is depleted, the first major chaperone to interact with nascent chains is the Hsp70 Ssb, in conjunction with the ribosome-associated complex (RAC) [16,21,25]. Ssb has dual functions as a ribosome-bound and cytosolic chaperone. In addition, it has a cytosol-only Hsp70 homolog Ssa [23]. Both chaperones have been shown to interact with Sup35[26–28]. We hypothesized that NAC deletion would impact the abundance, presence, or activity of the Hsp70s. We assessed total levels of Ssa and Ssb in each [*psi*-] and [*PSI*+] NAC deletion strain and found no difference relative to the wild type (S3A Fig).

We theorized that NAC deletion may increase the role of Ssb-RAC in cotranslational folding, as this protein complex is next in line to receive nascent chains following the NAC. This increased folding pressure upon Ssb-RAC as a result of NAC deletion might also localize a larger fraction of Ssb to the ribosome and nascent chains while titrating Ssb away from the available cytosolic pool. The titration would reduce the overall availability of cytosolic Ssb relative to Ssa, thereby creating a chaperone imbalance.

**Fig 4:**
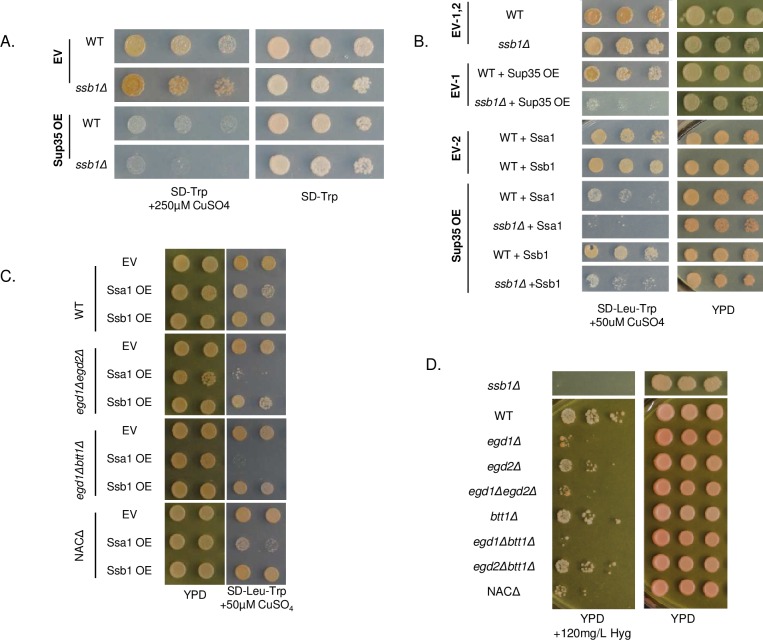
Chaperone balance is altered in NAC deletion strains. (A) WT and *ssb1Δ* strains were transformed with pCUP1-*SUP35* and spotted on plates containing 300μM copper to induce Sup35 overexpression. (B) WT and *ssb1Δ* strains were transformed sequentially with p314CUP-SUP35 and either p415GPD-*SSA1* or p415GPD-*SSB1*; transformants were spotted onto SD-Leu-Trp plates containing 50μM CuSO_4_. The *ssb1Δ* strain showed increased sensitivity to Sup35 overexpression in conjunction with Ssa1 overexpression. All strains on selective media are overexpressing Sup35. (C) WT and NAC deletion strains were first transformed with p314CUP-*SUP35*, then with either pRS415 (EV), p415GPD-*SSA1*, or p415GPD-*SSB1*. Transformants were spotted onto SD-Leu-Trp plates containing 50μM CuSO_4_ to induce non-toxic Sup35 overexpression. (D) WT and NAC deletion strains were spotted onto plates containing 120mg/L Hygromycin B. All strains pictured in panels A-D contain [*RNQ*+] and the strong variant of [*PSI*+].

We began by assessing the general effects of imbalanced Hsp70s in strains with intact NAC subunits. We challenged proteostasis by overexpressing Sup35 in a WT [*PSI*+] strain with endogenous Ssa expression and found that the cells grew as expected, with a moderate toxicity phenotype and consistent Sup35 expression (S3B Fig, Fig 4A). However, recapitulating an imbalance by deleting Ssb1 from this strain resulted in enhanced toxicity (Fig 4A). This indicates that cells are sensitive to chaperone balance in the presence of folding-challenged substrates. We then sought to verify that the balance of Ssb1 relative to Ssa1 was specifically affecting toxicity. To do so, we exacerbated the Hsp70 imbalance by overexpressing Ssa1 in WT and *ssb1Δ* strains. We again challenged proteostasis with overexpression of Sup35, but at a lower level than in the previous experiment. Slightly imbalanced Hsp70s (WT strain with SsaOE) led to poor growth, and severely imbalanced Hsp70s (*ssb1Δ* with Ssa1OE) led to pronounced toxicity (Fig 4B). Reintroduction of Ssb1 rescued the phenotype (Fig 4B). We concluded that cells are sensitive to the balance between Ssa and Ssb, and that the severity of the toxicity phenotype correlates with the severity of the imbalance.

We then sought to extend our analysis to the NAC deletion strains. As above, we overexpressed Ssa1 from a constitutive promoter in the WT and all NAC deletion strains; we observed no change in either growth or prion phenotype in the absence of Sup35 overexpression (S4 Fig and Fig 4C, YPD plates). However, when we subjected the same strains overexpressing Ssa to minor Sup35 overexpression, we observed a severe toxic phenotype in the *egd1Δegd2Δ* and *egd1Δbtt1Δ* strains (Fig 4C, selection plates) that mimicked the strong toxicity exhibited by *ssb1Δ* strains under the same conditions. Overexpression of Ssb1 did not cause cytotoxicity (Fig 4C). Ssa1 overexpression in the WT and single deletion strains did not perturb growth as severely (S5 Fig), possibly because the chaperone imbalance remained within a tolerable range. However, the toxicity in the *egd1Δegd2Δ* and *egd1Δbtt1Δ* strains indicates that Ssa1 overexpression pushes an already-imbalanced system into a highly detrimental state. This suggests that NAC deletion mimics Ssb deletion phenotypes despite the unchanged expression levels of all tested chaperones (S3 Fig), thereby supporting the concept of cellular localization changes and an imbalance of Ssb relative to Ssa.

We next questioned whether NAC deletion could phenocopy a chaperone imbalance in the absence of Sup35 overexpression. We tested the growth of [*PSI*+] NAC deletion strains under conditions that are disadvantageous to the *ssb1Δ* strain. We spotted the NAC deletion strains onto plates containing 120μg/L HygromycinB (HygB), as it is known that *ssb1Δ* yeast are sensitive to the fungicide [29]. Though WT yeast grew on HygB plates, several of the NAC deletion strains, including *egd1Δegd2Δ* and *egd1Δbtt1Δ*, exhibited poor growth consistent with depleted Ssb (Fig 4D). The growth similarities between NAC and Ssb deletion strains support the possibility that the NAC-induced chaperone imbalance may be altering the functionality of Ssb in ways that mimic its deletion.

### NAC deletion relocalizes Ssb to nascent polypeptides and away from prion aggregates

**Fig 5:**
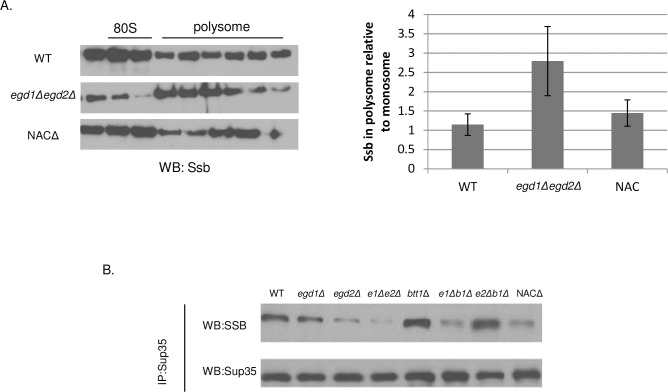
NAC deletion relocalizes Ssb to nascent polypeptides and away from prion aggregates. (A) Fractions from the monosome and polysome peaks of ribosome profile experiments were TCA precipitated prior to SDS-PAGE and Western blotting. More Ssb comigrated with polysomes in the *egd1Δegd2Δ* strain relative to the WT or to the whole-NAC deletion. Western blots are of the sucrose gradient fractions that contained the monosome and polysome peaks and are representative images from five independent experiments. Quantifications are from five independent experiments, and data are represented as mean ± SEM. See Materials and Methods for full computational details. (B) WT and NAC deletion strains were subjected to co-immunoprecipitation with an anti-Sup35 antibody. Equal amounts of Sup35 were immunoprecipitated across all strains. “Total” and “unbound” fractions were collected and no differences in Ssb protein expression levels were apparent (S3 Fig) Western blots are representative images from three independent experiments. All strains utilized contained [*RNQ*+] and the strong [*PSI*+] variant.

Though some NAC deletion strains exhibited phenotypes related to Ssb depletion, all strains exhibited WT levels of all tested chaperones (S3 Fig). We hypothesized that NAC subunit deletion led to alterations in Ssb localization and availability due to an additional requirement for Ssb at the ribosome. We reasoned that the loss of NAC subunits would cause Ssb to assist in the folding activities that were typically controlled by the NAC. To test this, we returned to our ribosome profile analysis (Fig 2C) and assessed the proteins present in the peak fractions. We theorized that more Ssb would be present in the polysome fractions of the *egd1Δegd2Δ* strain relative to the WT, because folding-challenged nascent chains would require more extensive cotranslational interactions with Ssb. We probed for the presence of Ssb in the polysome and monosome peaks and normalized to the amount of ribosomal protein Rpl3 detected in the same peaks (Fig 5A). The amount of Ssb in the polysome fractions was indeed increased in the *egd1Δegd2Δ* deletion strain relative to the WT, indicating a greater proportion of Ssb comigrating with polysomes. Thus, the localization of Ssb is altered in the NAC deletion strains relative to the WT, potentially modulating nascent Sup35 folding and related cytotoxicity.

To further probe the theory of Ssb relocalization, we sought to assess Ssb binding to aggregated Sup35. We hypothesized that an increase of Ssb binding to nascent chains would lead to a corresponding decrease in the pool of available (non-polysome bound) Ssb. This would cause decreased Ssb binding to Sup35 aggregates. We performed co-immunoprecipitation experiments in [*PSI*+] cells using a Sup35 antibody and probed for the presence of Ssb1/2. We found that the amount of Ssb1/2 that co-immunoprecipitated with Sup35 was reduced in several of the NAC deletions (Fig 5B), with the effect being most pronounced in *egd1Δegd2Δ* (50.0% reduction in co-immunoprecipiation, ±8.1%). The steady state levels of Ssb1/2 were not reduced in the NAC deletion strains (S3 Fig); thus, the decreased interactions with Sup35 represent a change in the binding between Ssb and its pool of post-translational substrates. This result, in combination with the enhanced presence of Ssb at translating ribosomes, indicates that Ssb localization shifts as a consequence of NAC subunit deletion.

### NAC deletion causes [*PSI*+] to resist curing by Hsp104 overexpression

We hypothesized that the imbalanced Hsp70s may impact other molecular chaperones in the NAC deletion strains. Ssa and Ssb both interact with the disaggregase Hsp104, which is required for the propagation of all but one of the known yeast prions [19,30]. We found no observable difference in steady state levels of Hsp104 (S3 Fig). Hsp104 activity was also not different between the WT and NAC deletion strains by thermotolerance tests (S6 Fig). Therefore, the observed toxicity rescue was not due to a change in presence or functionality of Hsp104.

Inhibition of Hsp104 is thought to cure prions by preventing fiber fragmentation, which impairs inheritance of seeds [19]. Overexpression of Hsp104 specifically cures [*PSI*+], and its curing ability is influenced by the Hsp70s. Ssb overexpression in conjunction with Hsp104 overexpression promotes loss of [*PSI*+], while Ssa1 overexpression prevents Hsp104-mediated curing [23,31,32]. Therefore, we predicted that Hsp104 curing efficiency may be altered in the NAC deletion strains due to the imbalance of Ssa and Ssb.

**Fig 6:**
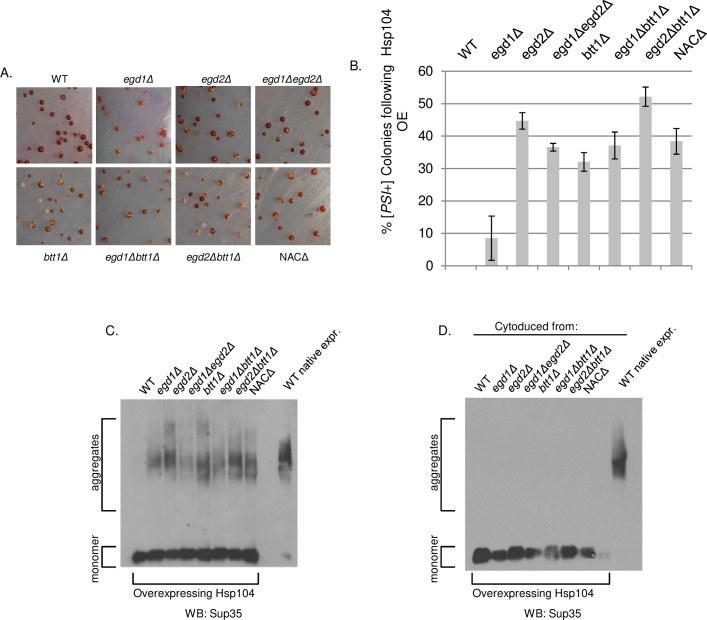
NAC deletion causes [*PSI*+] to resist curing by Hsp104 overexpression. WT and NAC deletion strains were transformed with p426GPD-*HSP104* to overexpress Hsp104 and then grown for 4 days at 30°C. (A) Representative images from transformation plates. Strains were originally strong [*PSI*+]; red color denotes curing of the [*PSI*+] prion. (B) Six independent transformations of NAC strains with p426GPD- *HSP104* were plated on selective media and [*PSI*+] status was quantified via phenotypic analysis of each colony. Data are represented as mean ± SEM. (C) Five colonies per NAC deletion strain, plus WT controls, were picked from transformation plates and grown 16hrs in selective media. SDD-AGE analysis shows the stability of the [*PSI*+] prion in the NAC deletion strains, but not the WT, upon overexpression of Hsp104. (D) Following cytoduction of [*PSI*+] from NAC deletion strains into a WT background, cytoductants did not retain the ability to resist curing by Hsp104 overexpression.

Hsp104 overexpression efficiently cures the [*PSI*+] prion, an effect that is inhibited by simultaneous Ssa overexpression. We wondered whether the Hsp70 imbalance in the NAC deletion strains would mimic this phenotype. We transformed each of the NAC deletion and WT strains with a plasmid that constitutively overexpresses Hsp104 and verified that Hsp104 levels were increased while not altering the growth of the cells or amount of Sup35 expressed (S7 Fig). We then phenotypically characterized the transformants by streaking them onto rich media. We again took advantage of the red/white colormetric assay that allowed us to track the presence of the yeast prion: white colonies harbor Sup35 aggregates while red colonies have been cured of [*PSI*+]. Surprisingly, all NAC deletion strains (with the exception of *egd1Δ*) were resistant to Hsp104-mediated curing of [*PSI*+], as a significant proportion of their colonies remained white on rich media on first pass (Fig 6A and 6B). This result was confirmed by performing an SDD-AGE assay to visualize the SDS-resistant Sup35 aggregates. Following continued growth with Hsp104 overexpression, aggregated Sup35 was still present in the NAC deletion strains but not the wild type (Fig 6C). This demonstrated that prion curing was less efficient in strains where NAC depletion was affecting chaperone balance and mimicked Ssa1 overexpression.

We then hypothesized that a heritable change in [*PSI*+] conformation or structure may have created Sup35 aggregates that were poor Hsp104 binding partners. These altered aggregates may thus be resistant to refolding by Hsp104 activity independent of a chaperone imbalance. We performed cytoduction experiments to test the curing of prions from NAC deletion strains in a WT genetic background [33]. We transferred Sup35 aggregates from WT and NAC deletion strains into wild type [*psi*-] strains by cytoplasmic transfer so that the resulting yeast were genetically WT but contained [*PSI*+] from the cohort of NAC deletion strains. We then induced Hsp104 overexpression and found that all of the cytoduced strains were cured as efficiently as WT (Fig 6D, S8A Fig). Thus, the heritable Sup35 aggregate structure was not the cause of differential Hsp104 curing; rather, the curing resistance exhibited by the deletion strains was a consequence of the genetic disruption of the NAC.

As the NAC deletion strains exhibited resistance to curing by Hsp104 overexpression, we wondered if they would also resist curing by Hsp104 inactivation. To test this, we passaged the NAC deletion strains on media containing 5mM guanidine hydrochloride (GdnHCl), a strong inhibitor of Hsp104 [34], which cures all known yeast prions. The WT and NAC deletion strains demonstrated equal curability on GdnHCl plates (S8B Fig). Taken together, these results suggest that the differential effects of the NAC interactions between Hsp104 and Sup35 are related the activity of co-chaperones.

### NAC deletion strains are resistant to general protein misfolding

**Fig 7:**
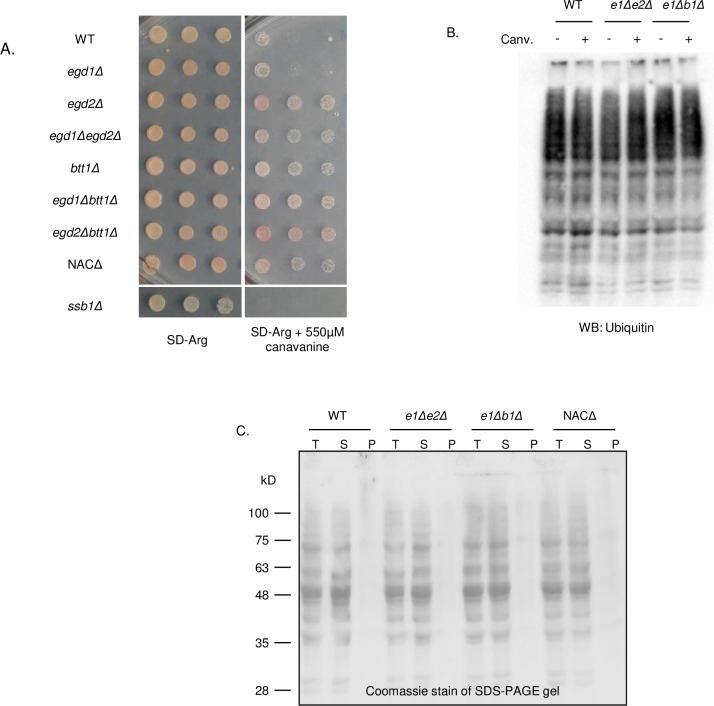
NAC deletion strains are able to resist protein misfolding without inducing stress response. (A) WT and NAC deletion strains were spotted on SD-Arg plates containing 550μM canavanine to induce global protein misfolding. (B) The total ubiquitinated proteins in WT, *egd1Δegd2Δ*, and *egd1Δbtt1Δ* are unchanged in both the presence and absence of canavanine. (C) Total aggregated protein was assessed via a solubility assay followed by SDS-PAGE and Coomassie staining.

Given the toxicity rescue phenotype resulting from loss of NAC subunits and altered chaperone activity, we considered that NAC deletion may bring about a broader modification to proteostasis and the cellular response to protein misfolding. First, we probed the ability of the NAC deletion strains to manage global protein misfolding. We challenged cells with canavanine, an arginine analog that induces misfolding [35], and found that most NAC deletion strains were able to survive high levels of the compound (Fig 7A), indicating that loss of NAC subunits allows cells to better tolerate the adverse effects of misfolding.

To determine how NAC deletion strains tolerate elevated protein misfolding, we asked whether misfolded proteins were differentially acted upon by protein quality control machinery in the NAC deletion strains. We questioned whether the NAC deletion strains resist canavanine-induced misfolding due to increased activity of the ubiquitin-proteasome system (UPS). We assessed the presence of ubiquitinated species in the NAC deletion strains in the presence and absence of canavanine and found no differences between the WT and the most stabilized NAC deletion strains (Fig 7B). Additionally, challenging the ubiquitin-proteasome system with heat stress or the proteasome inhibitor MG132 showed no differences between the WT and the NAC deletion strains (S6 Fig and S9A Fig). Thus, cellular viability in the presence of canavanine is not related to increased protein degradation as mediated by the UPS.

We considered the possibility that NAC deletion strains may package misfolded proteins into insoluble aggregates, rendering them nonfunctional but nontoxic. We performed solubility assays to visualize total protein aggregation in the NAC deletion strains, and observed no changes relative to the wild type (Fig 7C, S9C Fig). Therefore, we concluded that there is no gross global difference in the way proteins are packaged or degraded in the NAC deletion strains relative to the WT, indicating that the toxicity rescue phenotype is not related to enhanced stress response or turnover of misfolded proteins. Rather, like the prion-dependent effect, the chaperone imbalance renders cells generally resistant to misfolded proteins.

## Discussion

### Nascent polypeptides are functionally connected to cytosolic chaperones

**Fig 8:**
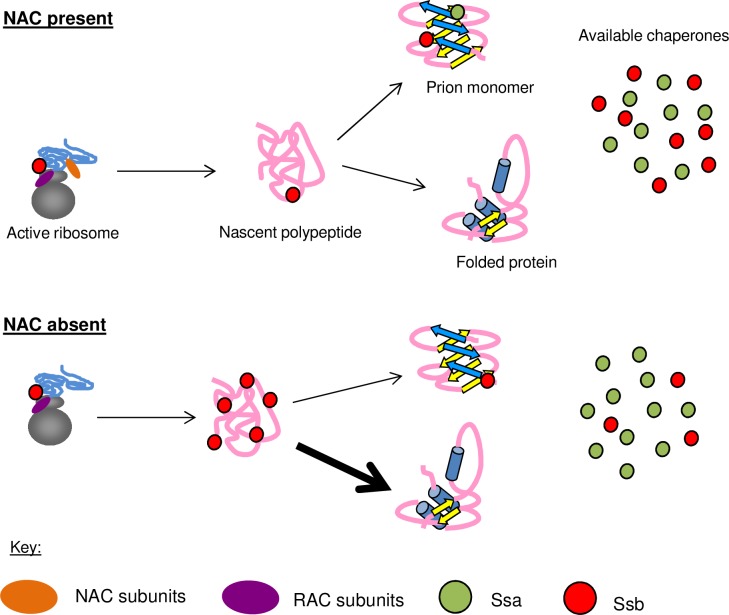
NAC subunits affect the yeast chaperone network by altering chaperone pools. In the presence of the NAC, RAC-Ssb receives nascent polypeptides from the NAC and assists with their folding. Both Ssa and Ssb bind to aggregated Sup35 and affect its ability to form and propagate the [*PSI*+] prion. When NAC subunits are deleted, Rac-Ssb becomes the first complete chaperone system to interact with nascent polypeptides. The unfolded state of these proteins requires more extensive interaction with RAC-Ssb, thereby sequestering cytosolic Ssb to the ribosome. Thus, less cytosolic Ssb is available to bind to monomeric or aggregated Sup35. This would lead to a relative increase in Ssa1 binding to Sup35 aggregates, which inhibits efficient monomer joining and reduces the ability of Hsp104 to cure the prion.

Here, we show that the NAC affects the localization and activities of other molecular chaperones. Our model (Fig 8) suggests that deletion of the α- and β-NAC subunits cause relocalization of the Hsp70 Ssb away from the available pool of cytosolic chaperones and to translating ribosomes, creating an imbalance that mimics Ssb deletion phenotypes. This depletion of Ssb from Sup35 aggregates serves to change the interaction of the [*PSI*+] prion with other chaperones, including Hsp104, which is less able to efficiently cure [*PSI*+] in the NAC deletion backgrounds. The deletion of the NAC does not impact expression levels for any of the proteins examined in this study (S3 Fig); thus, the observed phenotypes are due to changes in localization and functionality. These chaperone modifications correspond to an alteration in the aggregation pattern of the [*PSI*+] prion and the reduced ability of newly synthesized protein to join pre-existing aggregates. This alteration can be beneficial when cells are challenged with a toxic prion, presumably due to more active engagement of chaperones with nascent polypeptide chains where there is a high risk for misfolding [16]. We suggest a model of cotranslational folding that recruits cytosolic chaperones to nascent polypeptides in a manner that can rescue the toxicity associated with proteins prone to misfolding. Additional mechanistic studies will be necessary in order to determine the extent of Ssb activity on nascent chains in response to NAC deletion and how Ssb may differentially respond to the presence or absence of NAC subunits.

### Deletion of NAC subunits increases pressure on other cotranslational chaperones

The toxicity rescue effect of NAC subunit deletion was a surprising result. Deletion of cotranslational folding factors would not be expected to rescue toxicity associated with a prion-forming protein. We propose that deletion of NAC subunits creates an environment where the ribosome-associated complex and Ssb (RAC-Ssb) are the first fully-functional chaperones that interact with nascent polypeptides. Nascent polypeptides encountering the RAC-Ssb system in the NAC deletion strains are presumably less protected or folded than in WT cells. The RAC-Ssb system, compensating for loss of NAC subunits, might retain Ssb on nascent polypeptides, thereby reducing free Ssb elsewhere in the cell.

### NAC subunits may act independently upon partial deletion of the complex

Interestingly, deletion of the whole NAC did not recapitulate all of the phenotypes associated with the double deletions (summarized in S1 Table). In particular, whole-NAC deletion did not rescue [*PSI*+]-related Sup35 overexpression toxicity. Deletion of the entire NAC may exacerbate the chaperone imbalance in such a way that leads to a harmful depletion of the cytosolic pool of Ssb. However, in other cases, the triple NAC deletion did mimic phenotypes associated with the double deletions. For example, NAC deletion confers resistance to hygromycin B and increases resistance to Hsp104-mediated curing. This leads us to hypothesize that there is a function for each of the NAC subunits that can persist independent of the complex. For example, hygromycin B resistance is demonstrated by every strain that has a deletion of *EGD1*, indicating that this subunit may be particularly important in modulating the interaction between nascent chains and molecular chaperones. Further, in the NAC deletion strains, any subunits that remain in the cell might act independently on nascent chains or upon misfolded cytosolic proteins. The precise function of all of the NAC subunits remains unclear and we look forward to future studies that will shed light on this dynamic complex.

### NAC deletion changes chaperone localization, but not functionality

The ability of NAC deletion strains to mimic Ssb deletion phenotypes led us to question Ssb functionality in a NAC-depleted background. However, we have demonstrated that Ssb deletion is toxic in the presence of Sup35 overexpression, indicating that chaperone function is necessary. Further, perturbations that prevent Ssb association with the ribosome have been shown to enhance yeast sensitivity to Sup35 overexpression [36]. We suggest that the toxicity rescue observed in the NAC deletion strains is due to a shift in Ssb activity to nascent proteins. This change in localization and/or activity leads to a decrease in available Ssb relative to available Ssa.

Though we suggest an increase in Ssb localization to nascent polypeptides as a result of NAC deletion, we did not observe the distribution of Ssa to be affected. Ssa does not have an established role in cotranslational folding and is not ribosome-associated. Thus, in NAC deletion backgrounds in which Ssb becomes relocalized, the shift in the amount of available cytosolic Ssb relative to Ssa creates local imbalances between the Hsp70s at the ribosome, in the unbound cytosolic pool, and at prion aggregates. Thus, these strains can simultaneously exhibit phenotypes mimicking Ssa depletion (reduced de novo [*PSI*+] formation, S2 Fig), Ssa overexpression (resistance to Hsp104-mediated curing, Fig 6), Ssb deletion (sensitivity to HygB, Fig 4), and Ssb overexpression (prion toxicity rescue, Fig 1). This spectrum of effects suggests that neither Ssa nor Ssb has inhibited activity in the NAC deletion strains.

We were surprised that Ssa1 overexpression resulted in a toxicity phenotype in conjunction with slight Sup35 overexpression in [*PSI*+] NAC deletion strains. We hypothesize that Ssa overexpression sequesters an essential cofactor or substrate from Ssb; for example, an Hsp40 or a nucleotide exchange factor, which would in turn reduce Ssb’s folding capabilities. In the NAC deletion strains, the enhanced requirement of Ssb to fold nascent polypeptides would cause any perturbation of Ssb to be harmful to proteostasis and cellular health. Future studies will examine the competition between Hsp70s for multiple cofactors, a relationship that is not fully understood [37,38].

### Ssb relocalization prevents new monomer from joining and rescues [PSI+]-associated toxicity

Titrating Ssb away from the free pool of molecular chaperones has several effects on the cell and on the [*PSI*+] prion. Increasing the contact between Ssb and nascent polypeptides may cause Ssb to interact earlier with unfolded or misfolding Sup35. This would in turn inhibit the ability of nascent Sup35 to efficiently join aggregates, as Ssb overexpression is known to promote loss of [*PSI*+] [23]. In the most extreme case, the *egd1Δegd2Δ* strain, this joining defect manifests as fractured Sup35-GFP aggregates as viewed microscopically. This reorganization of aggregates may either release or reduce interaction with cofactors that are toxically sequestered during normal amyloid formation [7], leading to the rescue phenotype exhibited most strongly by the *egd1Δegd2Δ* strain.

It is likely that other NAC deletion strains undergo similar chaperone reorganization, but to a lesser extent depending on which NAC subunits remain to act upon nascent chains. This slight chaperone imbalance would lead to weaker phenotypes that evade detection. For example, the *egd1Δbtt1Δ* strain does not show altered Sup35-GFP aggregation, yet exhibits a joining defect via solubility assays. This strain also rescues prion-related toxicity, albeit to a weaker extent than the *egd1Δegd2Δ* strain.

### Chaperone inhibition is a promising anti-disease mechanism for mammalian systems

The stable propagation of [*PSI*+] by nontoxic Sup35 aggregates indicates that NAC deletion, and the subsequent chaperone imbalance, slows the toxic addition of Sup35 monomer to existing aggregates. Retention of aggregates without toxicity has implications for mammalian protein misfolding disorders that are spread via oligomeric species [39]. By reducing monomer joining onto existing amyloid, NAC deletion is blocking a key step in the prion life cycle. The resistance to global protein misfolding induced by canavanine in the NAC deletion strains (Fig 7A) indicates that depletion of the NAC, and subsequent functional substitution by other chaperones, can protect cells against non-prion misfolding. Future studies are needed to determine the effects of NAC deletion on the propagation and stability of additional fungal prions and other amyloidogenic proteins. In the context of human disease, amyloid plaque formation may be slowed or stopped if a similar mechanism can be unveiled.

The fact that chaperone deletion can be beneficial to cells, even in the face of protein misfolding stress, is a counterintuitive result. However, there is a growing body of evidence to support chaperone inactivation as a mechanism via which disease progression may be slowed. Many of these studies have focused on cancer [40], but recent research has found that Hsp70 imbalance leads to increased aggregation of the Alzheimer’s-related protein tau [41] and that Hsp70 inhibition can promote tau clearance [42]. Taken together, these studies highlight the importance of chaperone balance on maintaining cellular health, and implicate genetic and pharmacologic inhibition of chaperones, Hsp70s in particular, as a promising therapeutic avenue.

Our work builds upon the recent discoveries that the NAC can delay protein aggregation and provide feedback to translation machinery [43], assist with general protein folding and ribosome biogenesis [21], and that individual subunits have distinct functionalities related to protein folding and rescue of aggregation [44]. Together with our findings regarding the role of NAC subunits in regulating chaperone balance, this research points to the NAC as a major component in the protein homeostasis network. The NAC’s known significance in yeast and its ubiquity in Eukarya should motivate further investigation this multifunctional and essential complex.

## Materials and Methods

### Yeast strains, plasmids, cultures, and transformations

Yeast were cultured and transformed using standard techniques [45]. All deletion strains were created from the same 74D-697 [*RNQ*+][*PSI*+] parent. Genetic deletions were made by replacing the coding regions of *EGD1*, *EGD2*, and *BTT1* with *KANMX4*, *loxP-HIS3MX6-loxP*, and *loxP-URA3MX-loxP*; knockouts were confirmed by auxotrophic markers and colony PCR. Strains and plasmids are available in the supplemental experimental procedures.

### Fluorescence microscopy

Cells expressing pCUP1-*SUP35NM-GFP* were grown overnight in SD-Ura and Sup35NM-GFP expression was induced by the addition of CuSO_4_ to a final concentration of 50μM. After 2 hours of induction, cells were washed twice in 1X PBS, resuspended in 1X PBS, transferred to a 12-well glass slide (Erie), and observed with an Olympus FV1200 laser scanning confocal microscope fitted with a 100x oil immersion objective.

### Prion manipulation

SDD-AGE and colorimetric assays were performed as previously described [46]. Antibodies utilized in this study are available in the supplemental experimental procedures. Cytoduction was performed similarly to previous studies [47] to transfer a medium strain of [*RNQ*+] into [*rnq*-][*psi*-] strains (S2 Table). Putative cytoductants were selected on SGly-Ura and examined for accuracy by assessing auxotrophic markers. Prion transfer was confirmed by color on YPD and growth on SD-Ade plates. Haploid cytoductants retained the WT nucleus of the recipient strain and the strong [*PSI*+] prion aggregates of the donor strain.

### Ribosome profiling and quantification

Ribosome fractions were collected as previously described [48]. Protein precipitation of fractions was performed by TCA precipitation, followed by SDS-PAGE and Western blotting for the presence of Ssb and Rpl3. Quantification was performed with ImageJ, and background signal was subtracted from each measurement. The detected protein in the polysome fractions was divided by the detected protein in the monosome fraction as a way of controlling for the total amount of detected Ssb bound to ribosomes. We performed this analysis for Ssb, and scaled the quantification by the same method for the ribosomal protein Rpl3. This controlled for the total amount of protein in the polysome versus the monosome and allowed us to quantify changes in Ssb relative to the total ribosomal protein. The fraction of polysome-associated Ssb was normalized to the amount of polysome-associated Rpl3 from the same blot for both WT and *egd1Δegd2Δ* in triplicate. Thus, the quantification metric was calculated in this manner: $\frac{\left(\frac{Ssb\;in\;polysome}{Ssb\;in\;monosome}\right)}{\left(\frac{Rpl3\;in\;polysome}{Rpl3\;in\;monosome}\right)}$

### Joining assay

Yeast strains transformed with pCUP1-*SUP35* were grown overnight in SD-Trp media. At t = 0, CuSO_4_ was added to the media to a final concentration of 50μM. Aliquots were removed at indicated time points post-induction and cells were washed, pelleted, and frozen in liquid N_2_ prior to use. Cell lysis and protein extraction was adapted from the ball mill method [49].

### Co-Immunoprecipitation

Yeast strains were grown overnight in 10ml YPD or SD media. Cells were lysed in buffer (50mM Tris pH 8, 150mM NaCl, 1mM EDTA, 0.2% Triton X-100, protease inhibitors) with acid-washed glass beads (Sigma) for 2x3 minutes in a multi-tube vortexer (Scientific Industries). Lysates were incubated with 1μl Sup35 antibody at 4°C for 2 hours, and 40ul of a 50% slurry of Protein G sepharose beads (GE) and lysis buffer was added and tubes were incubated at 4°C overnight. An unbound fraction was retained and beads were washed 3X in lysis buffer and resuspended in SDS-PAGE sample buffer as the bound fraction. Fractions were boiled in sample buffer for 5 minutes before SDS-PAGE (10% polyacrylamide) and Western blotting with enhanced chemiluminescence (G-Biosciences) and film (GeneMate). Bands were quantified with ImageJ and normalized to immunoprecipitated Sup35.

### Sedimentation assay

Yeast were cultured in rich media under homeostatic conditions. Cells were lysed in buffer (100mM Tris pH 7.5, 200mM NaCl, 1mM EDTA, 5% glycerol, protease inhibitors) with glass beads as described above. A “total” fraction was retained, and then lysates were centrifuged at 250,000xg in a TLA100 rotor in an Optima TLX Ultracentrifuge (Beckman Coulter). The supernatant was retained as the “soluble” fraction, and then the pellets were washed in lysis buffer and centrifuged again. Supernatant was discarded and the pellets were resuspended in a 1:1 ratio of lysis buffer to RIPA buffer (50mM Tris pH 7, 200mM NaCl, 1% Triton X-100, 0.5% Na deoxycholate, 0.1% SDS). Following SDS-PAGE, gels were stained with coomassie blue to visualize the total protein content of each fraction.

## Supporting Information

S1 Fig Screen for factors that reduced prion-related toxicity. (A) We performed a transposon screen with a mini-transposon (3XHA/lacZ URA3 (mTn3)) mutagenized library [51]. The library was linearized with NotI and transformed into 74-D694 [*PSI*+] yeast containing pRS315*CUP SUP35* for the copper-inducible expression of Sup35. This strain harbors the strong [*PSI*+] prion variant. Prion variants (also called prion strains) result from particular amyloid structures propagated by the prion-forming protein [52]. Transformants were plated onto selective media containing 350μM CuSO_4_ and 1,243 putative suppressors were recovered. Candidates were picked with inoculating loops and respotted on the same media and 319 true suppressors were confirmed. Remaining candidates were mated to WT 74D-694 strains and sporulated to identify tetrads. Haploid candidates were confirmed by mating type testing. Eight candidates were recovered with phenotypes genetically linked to the transposon insertion. (B) Additional NAC deletion strains do not rescue [*PSI*+]-associated toxicity when Sup35 is overexpressed. The *egd2Δbtt1Δ* and *btt1Δ* strains show poor growth on selective media containing 350μM CuSO_4_.
(TIF)

S2 Fig The [*PSI*+] prion is altered in NAC deletion strains. (A) A second disruption of the *EGD1* and *EGD2* genes (“clone 2”) displays a similar pattern of Sup35 aggregation as is shown in Fig 3A. (B) The 1 hour timepoint for the joining assay performed in Fig 3C. (C) The amount of Sup35-GFP induced by addition of CuSO_4_ to the culture medium was consistent between strains. Note the proportion of Sup35-GFP present in the “uninduced” lane, consistent with leaky expression from the CUP1 promoter [53]. (D) WT and NAC deletion strains were cured of all prions by three passages on media containing 5mM GdnHCl. Strains were cytoduced with the “medium” variant of [*RNQ*+] [47] and transformed with pEMBL-SUP35. Induction of [*PSI*+] was performed as previously described [54]. At least three independent experiments were performed and a minimum of 600 colonies were counted. Data are represented as mean ± SEM. * = p<0.07; ** = p<0.05. (E) Covering the *egd1Δ* deletion with a plasmid expressing Egd1 gene rescues [*PSI*+] induction to WT levels. Data are represented as mean ± SEM.
(TIF)

S3 Fig Deletion of NAC subunits does not globally alter protein expression levels. (A) Wild type and NAC deletion strains were grown overnight in YPD before lysis, SDS-PAGE, and Western blotting for the specified proteins. Expression levels of chaperone (Sis1, Ssb1/2, Hsp104) and prion-forming (Sup35, Rnq1) proteins is not changed as a result of NAC deletion. The vertical white bar in the Hsp104 blot indicates non-contiguous lanes of the same blot. (B) Sup35 expression was analyzed by Western blot of lysates of the WT and *ssb1Δ* strains from Fig 4A. The vertical white bar indicates non-contiguous lanes of the same blot.
(TIF)

S4 Fig Ssa1 is not toxic at steady-state levels of Sup35 expression. WT and NAC deletion strains were transformed with p415GPD-SSA1 for the overexpression of Ssa1. Overexpression of Ssa1 is not toxic without the concurrent overexpression of Sup35. All strains contain [*RNQ*+] and the strong [*PSI*+] variant; none of the strains are overexpressing Sup35.
(TIF)

S5 Fig Ssa1 overexpression can be toxic in conjunction with Sup35 overexpression. Spottings performed (as in Fig 4C) demonstrate that the effects of Ssa1 overexpression, in conjunction with Sup35 overexpression, are less toxic in the single NAC deletion strains than in the double deletions. All strains contain [*RNQ*+] and the strong [*PSI*+] variant, and the strains on selective media are overexpressing Sup35.
(TIF)

S6 Fig The thermotolerance of NAC deletion strains is unchanged. Strains (all [*RNQ*+] and strong [*PSI*+]) were grown in YPD at 30C and separated into 500μl fractions in glass culture tubes. Culture tubes were “pretreated” for 30 minutes at 37°C prior to heatshock to promote the induction of heat-responsive elements. A non-pretreated control (-P) was incubated at 30°C. Cultures were heat shocked at 50 degrees for the indicated number of minutes. NS = no shock. No differences were observed between the WT and deletion strains.
(TIF)

S7 Fig Hsp104 overexpression does not alter Sup35 expression or strain growth. The WT and NAC deletion strains were transformed with a plasmid to overexpress Hsp104, as described in Fig 6. (A) The amount of Hsp104 overexpression was consistent between the transformed strains, and substantially greater than the empty vector control grown in identical media. (B) The levels of expressed Sup35 was unchanged in the Hsp104-overexpressing strains. The empty vector (EV) control does not overexpress Hsp104. (C) Strains overexpressing Hsp104 were grown in selective media in a plate reader to monitor their growth over time. There were no differences between the WT and NAC deletion strains; thus, growth rate does not account for the changes in [PSI+] curing efficiency. Results are the averaged values of three experiments.
(TIF)

S8 Fig NAC deletion strains are only resistant to [*PSI*+] curing by Hsp104 overexpression. (A) As in Fig 6D, a WT strain was cytoduced with prion aggregates from WT or NAC deletion strains, and then transformed with a plasmid that overexpresses Hsp104. (B) [*PSI*+] WT and NAC deletion strains were spotted onto plates containing variable levels of GdnHCl (5mM plates are shown), which inactivates Hsp104. All strains demonstrated equal curability, as demonstrated by their red coloration.
(TIF)

S9 Fig NAC deletion strains do not demonstrate elevated stress responses. (A) WT and NAC deletion strains (all [*RNQ*+] and strong [*PSI*+]) were spotted onto plates containing the proteasome inhibitor MG132. The strains were not differentially affected by the stressor. (B) The induction of the unfolded protein response (UPR) was measured at baseline (uninduced) and upon addition of tunicamycin to culture medium (activated UPR). The NAC deletion strains did not show a differential ability to induce the UPR in response to misfolding stress. (C) The solubility assay from Fig 7C was repeated with concentrated lysates in order to visualize the insoluble (pellet) fraction. No differences were observed between the WT and NAC deletion strains, indicating that none of these strains show an increased or decreased accumulation of aggregated material.
(TIF)

S1 Table Summary of the phenotypes displayed by the NAC deletion strains. The *egd1Δegd2Δ* and *egd1Δbtt1Δ* strains were able to rescue prion–associated toxicity and had the most severe phenotypes in the remaining assays. Strains that showed variable phenotypes (e.g. *egd2Δ* could resist canavanine but not HygB) may harbor a slight chaperone imbalance that could not produce a detectable readout in our assays.
(PDF)

S2 Table Strains used in this study. (PDF)

S3 Table Plasmids used in this study. (PDF)

S4 Table Antibodies used in this study. (PDF)

S1 References Additional references for supporting information. (PDF)
