# Supplementary figures and images for: Prion-Associated Toxicity is Rescued by Elimination of Cotranslational Chaperones
Source: PLoS Genet. 2016 Nov 9;12(11):e1006431. doi: 10.1371/journal.pgen.1006431 (PMC5102407; doi:10.1371/journal.pgen.1006431)

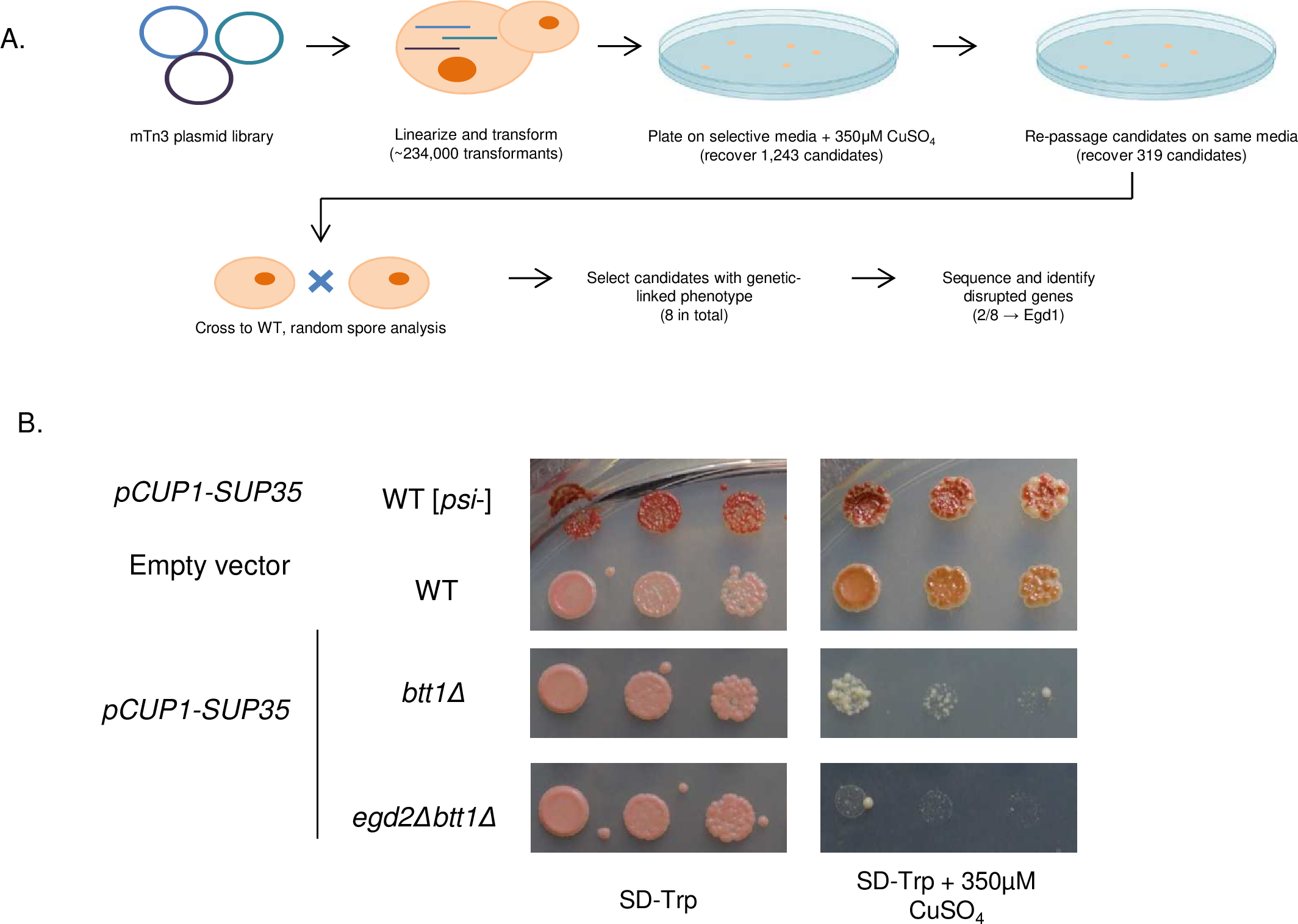

Supplement: S1 Fig — (A) We performed a transposon screen with a mini-transposon (3XHA/lacZ URA3 (mTn3)) mutagenized library [51]. The library was linearized with NotI and transformed into 74-D694 [PSI+] yeast containing pRS315CUP SUP35 for the copper-inducible expression of Sup35. This strain harbors the strong [PSI+] prion variant. Prion variants (also called prion strains) result from particular amyloid structures propagated by the prion-forming protein [52]. Transformants were plated onto selective media containing 350μM CuSO4 and 1,243 putative suppressors were recovered. Candidates were picked with inoculating loops and respotted on the same media and 319 true suppressors were confirmed. Remaining candidates were mated to WT 74D-694 strains and sporulated to identify tetrads. Haploid candidates were confirmed by mating type testing. Eight candidates were recovered with phenotypes genetically linked to the transposon insertion. (B) Additional NAC deletion strains do not rescue [PSI+]-associated toxicity when Sup35 is overexpressed. The egd2Δbtt1Δ and btt1Δ strains show poor growth on selective media containing 350μM CuSO4. (TIF) [file pgen.1006431.s001.tif]

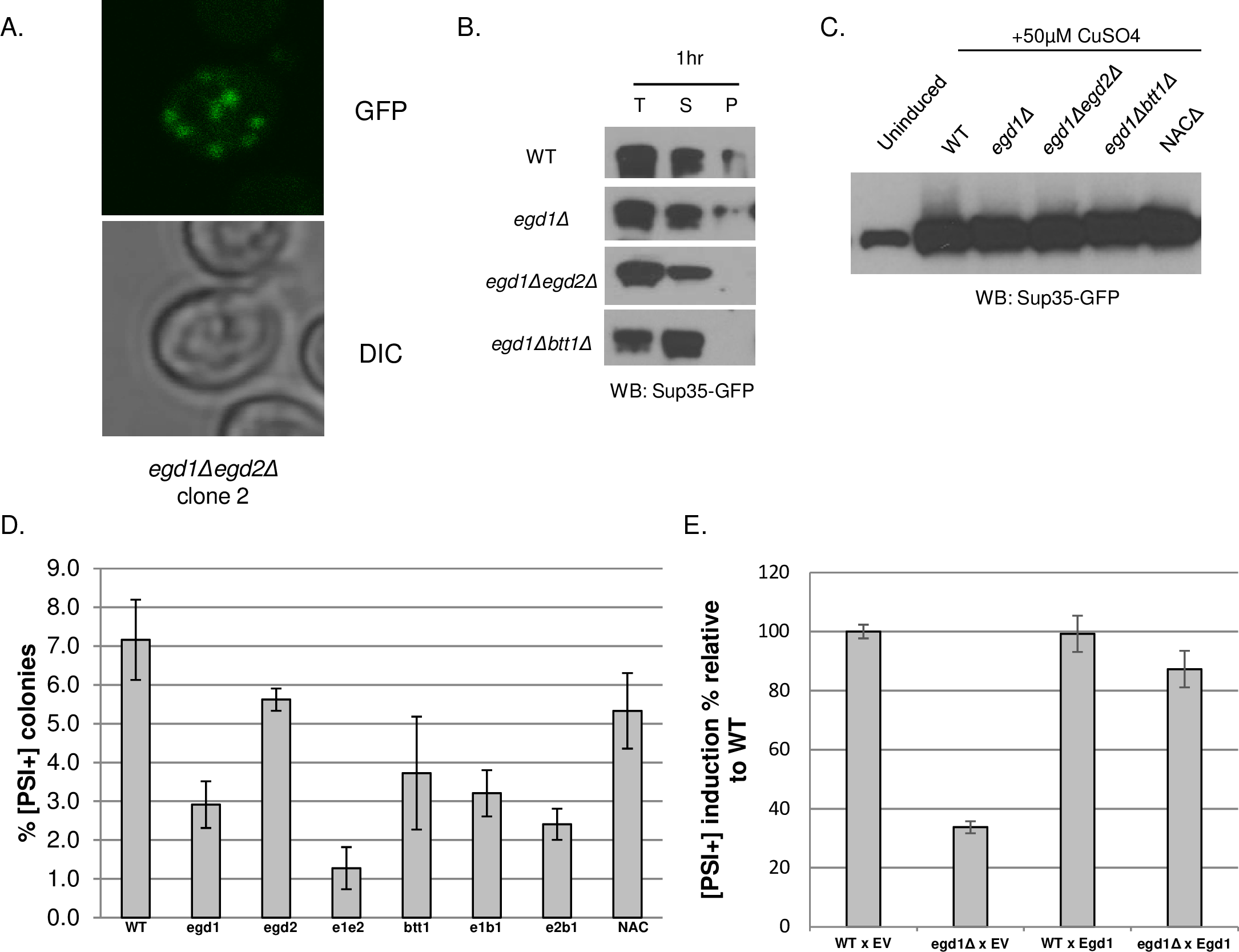

Supplement: S2 Fig — (A) A second disruption of the EGD1 and EGD2 genes (“clone 2”) displays a similar pattern of Sup35 aggregation as is shown in Fig 3A. (B) The 1 hour timepoint for the joining assay performed in Fig 3C. (C) The amount of Sup35-GFP induced by addition of CuSO4 to the culture medium was consistent between strains. Note the proportion of Sup35-GFP present in the “uninduced” lane, consistent with leaky expression from the CUP1 promoter [53]. (D) WT and NAC deletion strains were cured of all prions by three passages on media containing 5mM GdnHCl. Strains were cytoduced with the “medium” variant of [RNQ+] [47] and transformed with pEMBL-SUP35. Induction of [PSI+] was performed as previously described [54]. At least three independent experiments were performed and a minimum of 600 colonies were counted. Data are represented as mean ± SEM. * = p<0.07; ** = p<0.05. (E) Covering the egd1Δ deletion with a plasmid expressing Egd1 gene rescues [PSI+] induction to WT levels. Data are represented as mean ± SEM. (TIF) [file pgen.1006431.s002.tif]

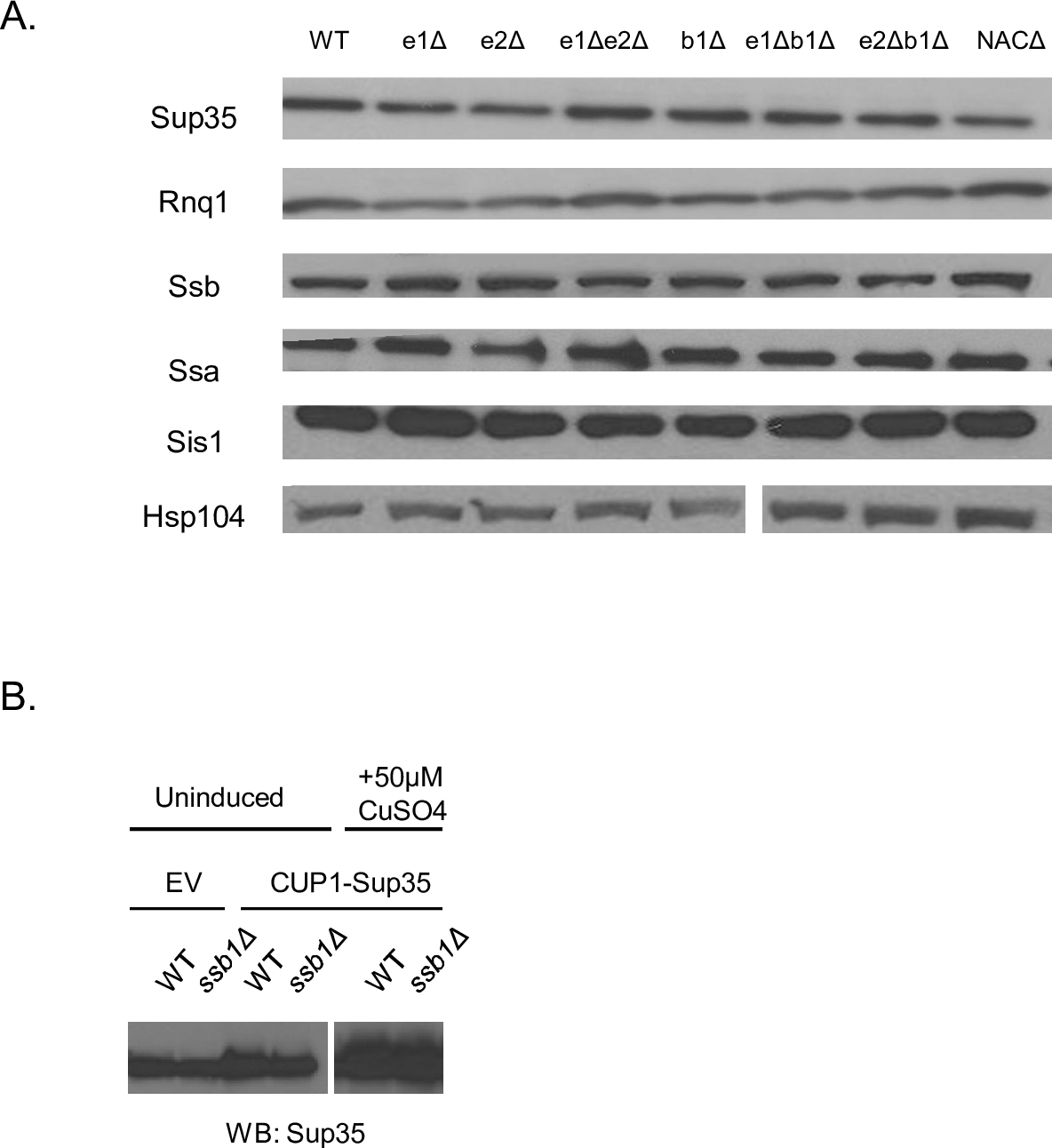

Supplement: S3 Fig — (A) Wild type and NAC deletion strains were grown overnight in YPD before lysis, SDS-PAGE, and Western blotting for the specified proteins. Expression levels of chaperone (Sis1, Ssb1/2, Hsp104) and prion-forming (Sup35, Rnq1) proteins is not changed as a result of NAC deletion. The vertical white bar in the Hsp104 blot indicates non-contiguous lanes of the same blot. (B) Sup35 expression was analyzed by Western blot of lysates of the WT and ssb1Δ strains from Fig 4A. The vertical white bar indicates non-contiguous lanes of the same blot. (TIF) [file pgen.1006431.s003.tif]

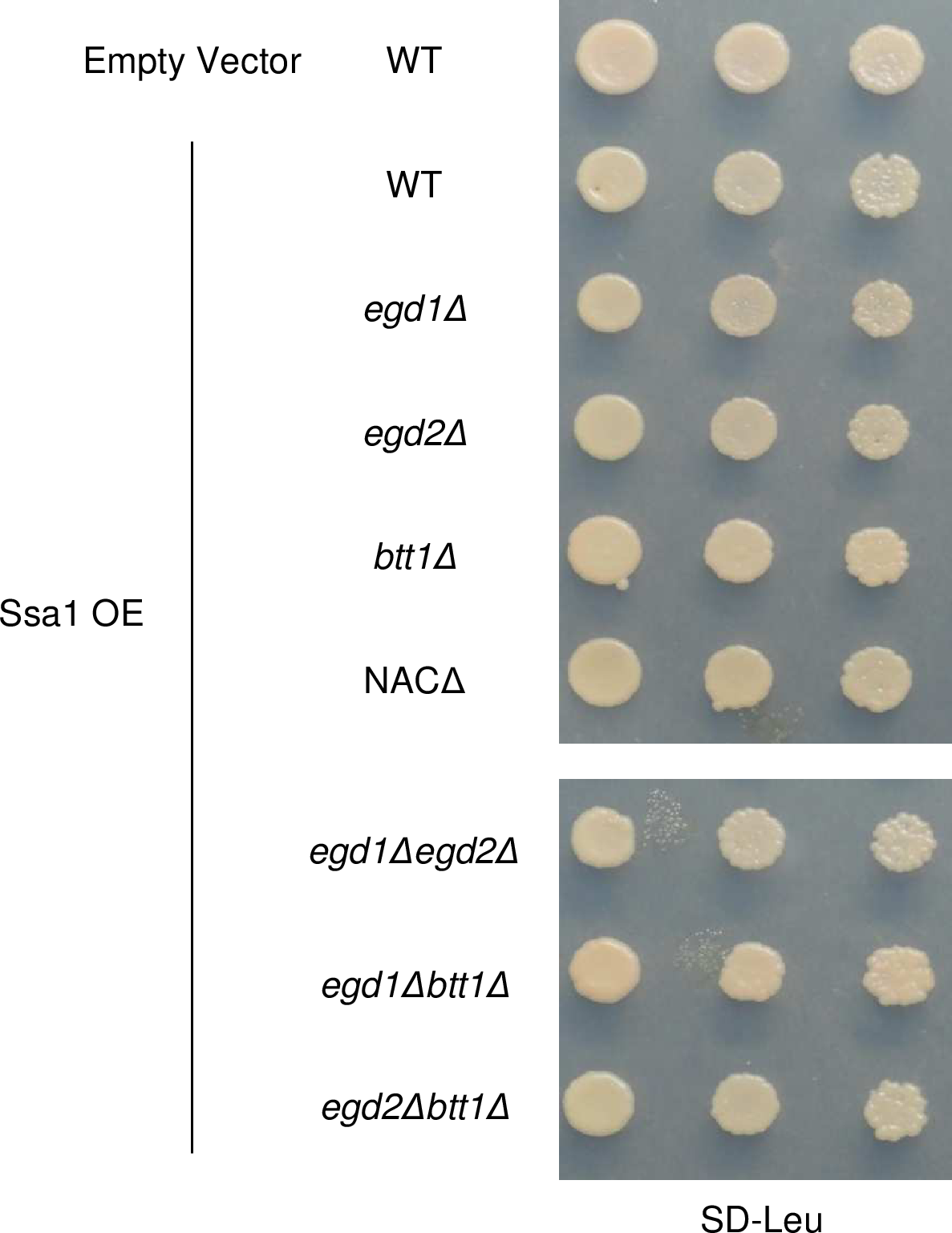

Supplement: S4 Fig — WT and NAC deletion strains were transformed with p415GPD-SSA1 for the overexpression of Ssa1. Overexpression of Ssa1 is not toxic without the concurrent overexpression of Sup35. All strains contain [RNQ+] and the strong [PSI+] variant; none of the strains are overexpressing Sup35. (TIF) [file pgen.1006431.s004.tif]

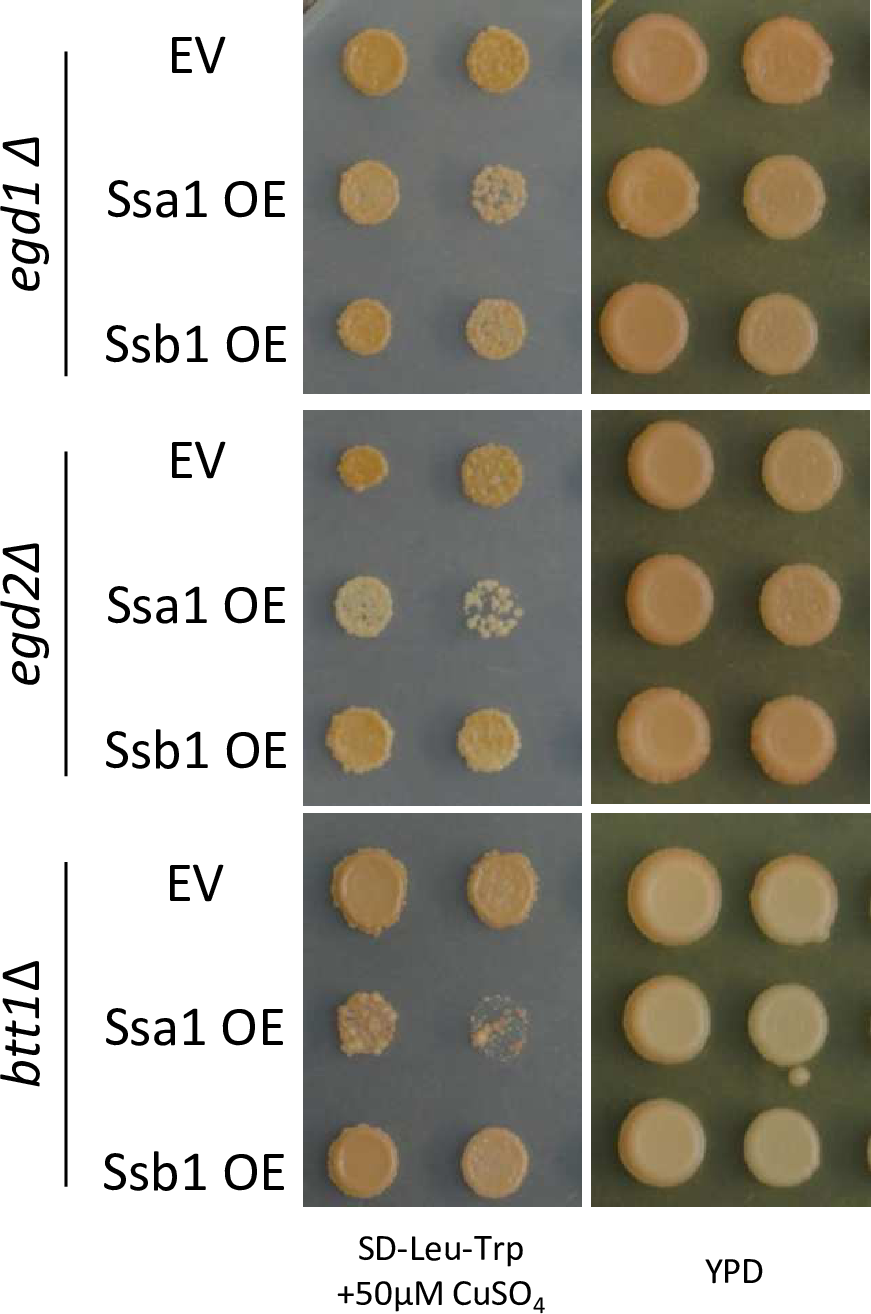

Supplement: S5 Fig — Spottings performed (as in Fig 4C) demonstrate that the effects of Ssa1 overexpression, in conjunction with Sup35 overexpression, are less toxic in the single NAC deletion strains than in the double deletions. All strains contain [RNQ+] and the strong [PSI+] variant, and the strains on selective media are overexpressing Sup35. (TIF) [file pgen.1006431.s005.tif]

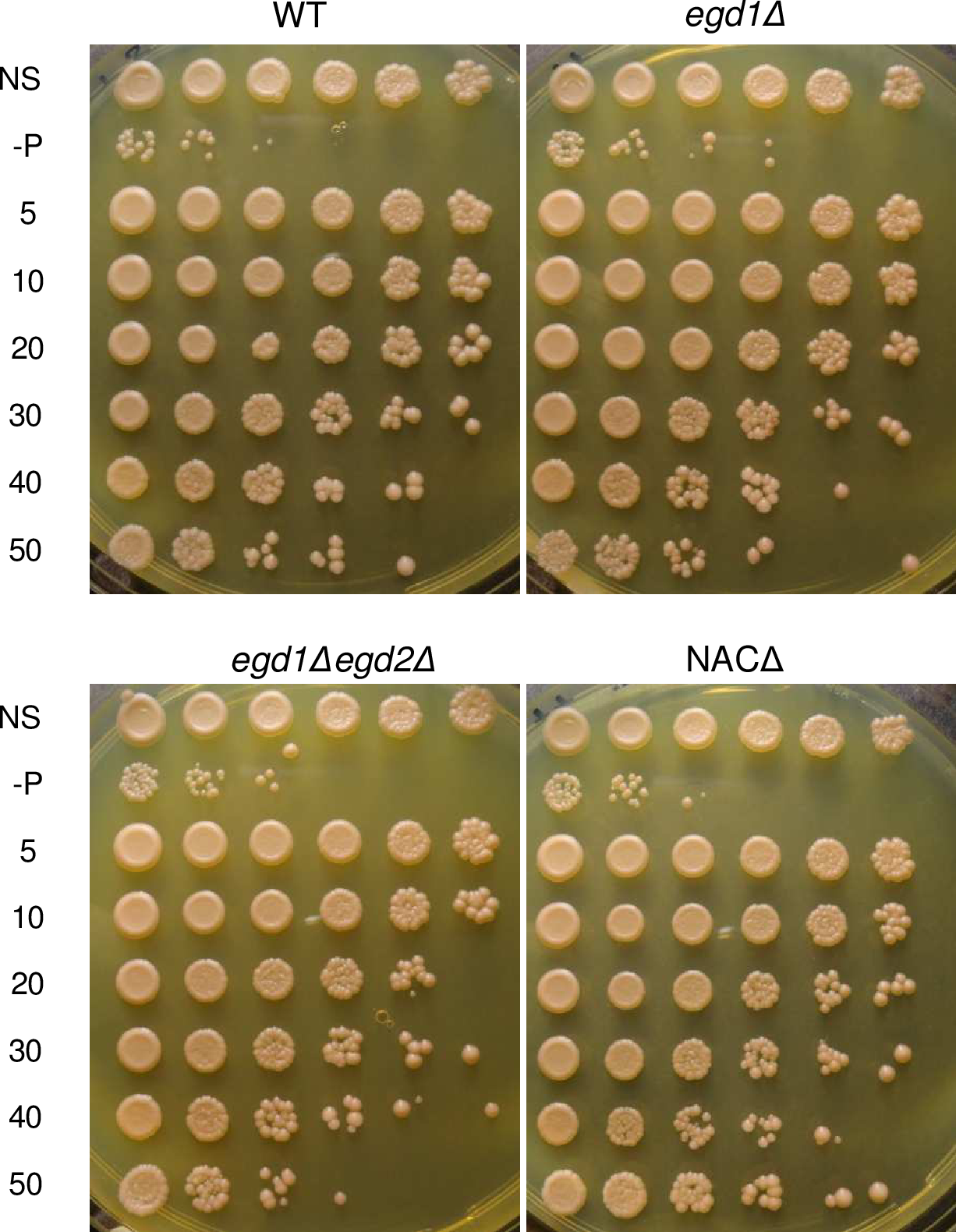

Supplement: S6 Fig — Strains (all [RNQ+] and strong [PSI+]) were grown in YPD at 30C and separated into 500μl fractions in glass culture tubes. Culture tubes were “pretreated” for 30 minutes at 37°C prior to heatshock to promote the induction of heat-responsive elements. A non-pretreated control (-P) was incubated at 30°C. Cultures were heat shocked at 50 degrees for the indicated number of minutes. NS = no shock. No differences were observed between the WT and deletion strains. (TIF) [file pgen.1006431.s006.tif]

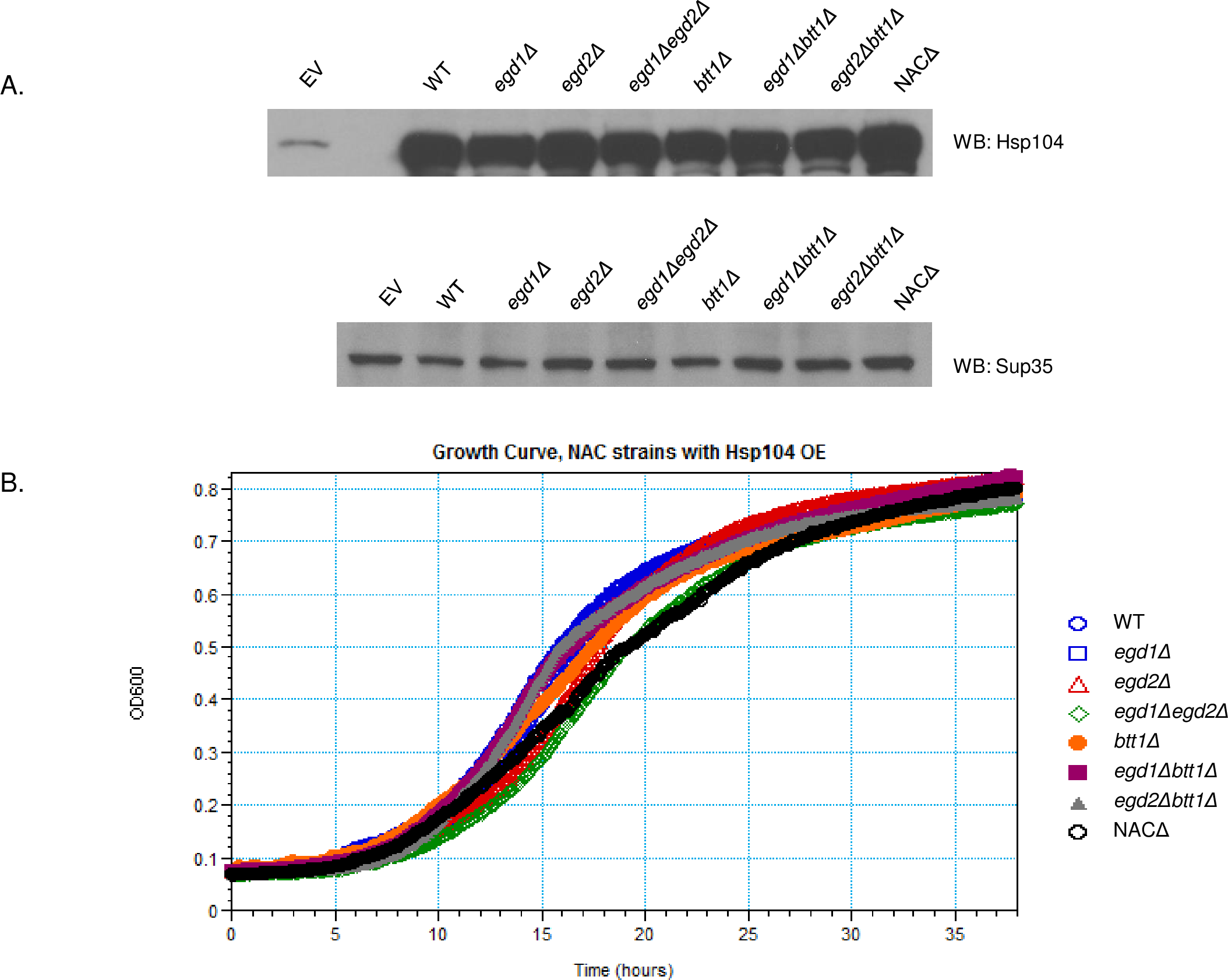

Supplement: S7 Fig — The WT and NAC deletion strains were transformed with a plasmid to overexpress Hsp104, as described in Fig 6. (A) The amount of Hsp104 overexpression was consistent between the transformed strains, and substantially greater than the empty vector control grown in identical media. (B) The levels of expressed Sup35 was unchanged in the Hsp104-overexpressing strains. The empty vector (EV) control does not overexpress Hsp104. (C) Strains overexpressing Hsp104 were grown in selective media in a plate reader to monitor their growth over time. There were no differences between the WT and NAC deletion strains; thus, growth rate does not account for the changes in [PSI+] curing efficiency. Results are the averaged values of three experiments. (TIF) [file pgen.1006431.s007.tif]

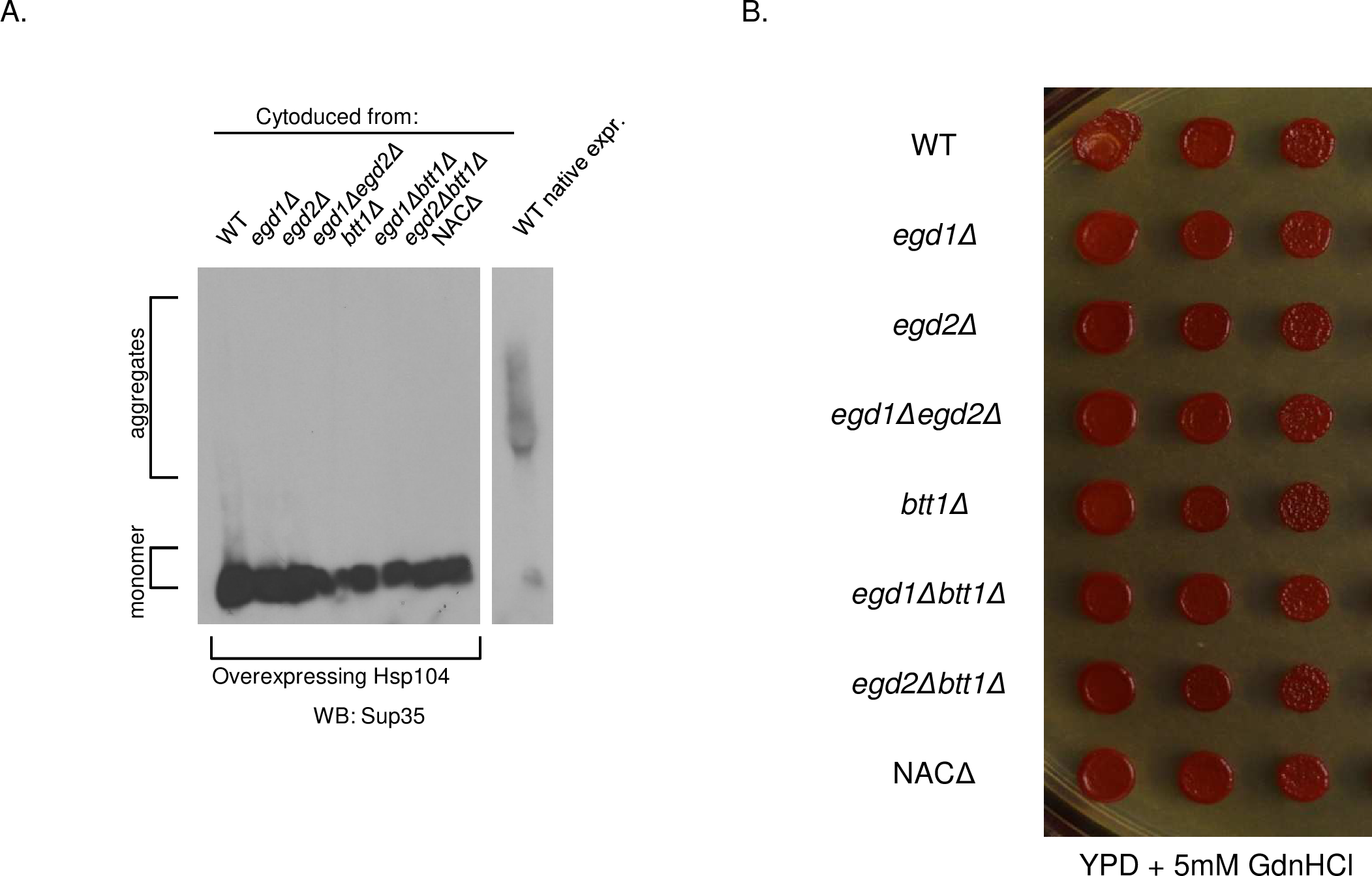

Supplement: S8 Fig — (A) As in Fig 6D, a WT strain was cytoduced with prion aggregates from WT or NAC deletion strains, and then transformed with a plasmid that overexpresses Hsp104. (B) [PSI+] WT and NAC deletion strains were spotted onto plates containing variable levels of GdnHCl (5mM plates are shown), which inactivates Hsp104. All strains demonstrated equal curability, as demonstrated by their red coloration. (TIF) [file pgen.1006431.s008.tif]

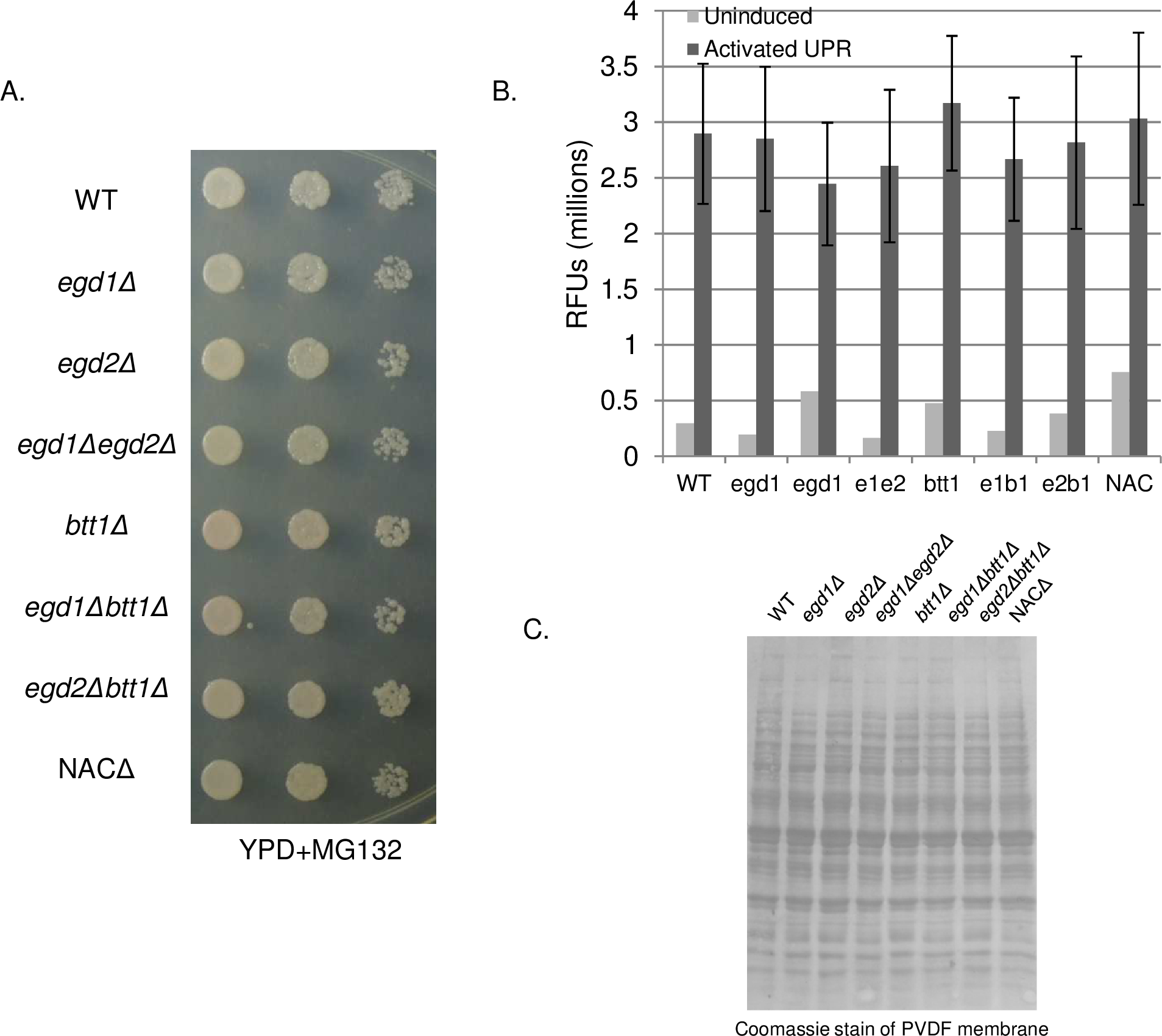

Supplement: S9 Fig — (A) WT and NAC deletion strains (all [RNQ+] and strong [PSI+]) were spotted onto plates containing the proteasome inhibitor MG132. The strains were not differentially affected by the stressor. (B) The induction of the unfolded protein response (UPR) was measured at baseline (uninduced) and upon addition of tunicamycin to culture medium (activated UPR). The NAC deletion strains did not show a differential ability to induce the UPR in response to misfolding stress. (C) The solubility assay from Fig 7C was repeated with concentrated lysates in order to visualize the insoluble (pellet) fraction. No differences were observed between the WT and NAC deletion strains, indicating that none of these strains show an increased or decreased accumulation of aggregated material. (TIF) [file pgen.1006431.s009.tif]
