# Supplementary material for: Prion-Associated Toxicity is Rescued by Elimination of Cotranslational Chaperones
Source: PLoS Genet. 2016 Nov 9;12(11):e1006431. doi: 10.1371/journal.pgen.1006431 (PMC5102407; doi:10.1371/journal.pgen.1006431)
Supplement: S1 Table — The egd1Δegd2Δ and egd1Δbtt1Δ strains were able to rescue prion–associated toxicity and had the most severe phenotypes in the remaining assays. Strains that showed variable phenotypes (e.g. egd2Δ could resist canavanine but not HygB) may harbor a slight chaperone imbalance that could not produce a detectable readout in our assays. (PDF) [file pgen.1006431.s010.pdf]

S1 Table

**Strain** *all [PSI+]*	**Robust toxicity rescue**	**Resists canavanine**	**Ssb binding to Sup35** *relative to WT*	**Sensitive to HygB**	**Sensitive to SsaOE**	**Resistant to Hsp104 curing**	**Altered Sup35 aggregates**	**Joining defect**
WT	*no*	*no*	X	*no*	*no*	*no*	*no*	*no*
egd1Δ	*no*	*no*	*unchanged*	YES	*no*	moderate	*no*	*no*
egd2Δ	*no*	YES	*n.s*	*no*	*no*	YES	*no*	*no*
egd1Δegd2Δ	YES	YES	reduced	YES	YES	YES	YES	YES
btt1Δ	*no*	YES	increased	*no*	*no*	YES	*no*	*no*
egd1Δbtt1Δ	YES	YES	reduced	YES	YES	YES	*no*	YES
egd2Δbtt1Δ	*no*	YES	*unchanged*	*no*	YES	YES	*no*	*no*
NACΔ	*no*	YES	*n.s*	YES	YES	YES	*no*	*no*
