# Supplementary material for: Prion-Associated Toxicity is Rescued by Elimination of Cotranslational Chaperones
Source: PLoS Genet. 2016 Nov 9;12(11):e1006431. doi: 10.1371/journal.pgen.1006431 (PMC5102407; doi:10.1371/journal.pgen.1006431)
Supplement: S2 Table — (PDF) [file pgen.1006431.s011.pdf]

S2 Table

Strain	Description	Reference
SL2598	74D-694; Strong [*PSI*+], MATa, ade1-14(UGA), trp1-289(UAG), his3Δ-200, ura3-52, leu2-3,112	[55]
1271	74D-694; Strong [*PSI*+], MATa, ade1-14 his 3Δ-200 trp 1-289 ura3-52 leu2-3,112 egd1D::KanMX4	This study
1147	74D-694; Strong [*PSI*+], MATα, ade1-14 his 3Δ-200 trp 1-289 ura3-52 leu2-3,112 egd2Δ::loxP	This study
1148	74D-694; Strong [*PSI*+], MATa, ade1-14 his 3Δ-200 trp 1-289 ura3-52 leu2-3,112 egd1Δ::KanMX4 egd2Δ::loxP – clone #1	This study
962	74D-694; Strong [*PSI*+], MATa, ade1-14 his 3Δ-200 trp 1-289 ura3-52 leu2-3,112 btt1Δ::HIS5	This study
966	74D-694; Strong [*PSI*+], MATa, ade1-14 his 3Δ-200 trp 1-289 ura3-52 leu2-3,112 egd1Δ::KanMX4 btt1Δ::HIS5	This study
1150	74D-694; Strong [*PSI*+], MATa, ade1-14 his 3Δ-200 trp 1-289 ura3-52 leu2-3,112 btt1Δ::loxP egd2Δ::loxP	This study
1152	74D-694; Strong [*PSI*+], MATa, ade1-14 his 3Δ-200 trp 1-289 ura3-52 leu2-3,112 egd1Δ::KanMX4 btt1Δ::loxP egd2Δ::loxP	This study
2352	74D-694; [*psi*-],mediuim [*RNQ*+], MATα, ade1-14, trp1-289(UAG), his3Δ-200, leu2-3,112, ρ0, kar1-Δ15	[47]
2090	74D-694; [*psi*-],medium [*RNQ*+] MATa, ade1-14, trp1-289(UAG), his3Δ-200, leu2-3,112, ρ0, kar1-Δ15	[47]
SL3014	74D-694; [*psi*-], MATα, ade1-14, his 3Δ-200 trp 1-289 ura3-52 leu2-3,112	[55]
1142	74D-694; Strong [*PSI*+], MATα, ade1-14, his 3Δ-200 trp 1-289 ura3-52, leu2-3,112, ssb1Δ::loxP	This study
1149	74D-694; Strong [*PSI*+], MATa, ade1-14 his 3Δ-200 trp 1-289 ura3-52 leu2-3,112 egd1Δ::KanMX4 egd2Δ::loxP – clone #2	This study
