# Supplementary material for: Prion-Associated Toxicity is Rescued by Elimination of Cotranslational Chaperones
Source: PLoS Genet. 2016 Nov 9;12(11):e1006431. doi: 10.1371/journal.pgen.1006431 (PMC5102407; doi:10.1371/journal.pgen.1006431)
Supplement: S3 Table — (PDF) [file pgen.1006431.s012.pdf]

S3 Table

Plasmid	Description and use	Reference
5162	pCUP-Sup35-GFP. Copper-inducible expression of Sup35-GFP.	This study
6244	pEMBL-Sup35. Overexpression of Sup35.	[56]
SL6435	pUKC815. Control for nonsense suppression assay. PGK1-LacZ fusion.	[20]
SL6436	pUKC817. Nonsense suppression assay. PGK1(TAA)LacZ fusion.	[20]
SL6437	pUKC818. Nonsense suppression assay. PGK1(TAG)LacZ fusion.	[20]
SL6438	pUKC819. Nonsense suppression assay. PGK1(TGA)LacZ fusion.	[20]
SL7280	p426GPD-Hsp104. Used for the overexpression of Hsp104.	[57]
6221	pCUP-Sup35NM-GFP. Copper-inducible expression of Sup35NM-GFP.	[55]
6765	p424GPD-Hsp104. Used for the overexpression of Hsp104.	This study
6391	p415GPD-Ssa1. Overexpression of Ssa1.	This study
6750	p415GPD-Ssb1. Overexpression of Ssb1.	This study
6751	P415GPD-Ssb2. Overexpression of Ssb2.	This study
EC1219	pRS316K-SSB1. Wild type Ssb1, “BBB.”	[29]
EC1327	pRS316K-SSA1. Wild type Ssa1, “AAA.”	[29]
EC1659	pRS316K-ABB. Chimera of Ssa1 and Ssb1.	[29]
EC1662	pRS316K-BAA. Chimera of Ssa1 and Ssb1.	[29]
EC1695	pRS316K-AAB. Chimera of Ssa1 and Ssb1.	[29]
EC1696	pRS316K-ABA. Chimera of Ssa1 and Ssb1.	[29]
EC1697	pRS316K-BAB. Chimera of Ssa1 and Ssb1.	[29]
EC1698	pRS316K-BBA. Chimera of Ssa1 and Ssb1.	[29]
