# Supplementary material for: Prion-Associated Toxicity is Rescued by Elimination of Cotranslational Chaperones
Source: PLoS Genet. 2016 Nov 9;12(11):e1006431. doi: 10.1371/journal.pgen.1006431 (PMC5102407; doi:10.1371/journal.pgen.1006431)
Supplement: S4 Table — (PDF) [file pgen.1006431.s013.pdf]

S4 Table

Antigen	Production notes	Type	Dilution	Source
Sup35	Peptide, a.a.s 137-151	Rabbit polyclonal	1:1,500	True lab
Rnq1	Full-length Rnq1	Rabbit polyclonal	1:1,000	True lab
Ssb1/2	C-terminal 80 a.a.s	Rabbit polyclonal	1:2,000	Craig lab [58]
Rpl3	IgG2b, ascites	Mouse monoclonal	1:2,500	Warner lab [59]
Hsp104	Carboxy terminus	Rabbit polyclonal	1:3,000	Glover lab [60]
Sis1	Peptide, a.a.s 339-352	Rabbit polyclonal	1:5,000	Cosmo Bio p89
GFP	N-terminal peptide	Mouse monoclonal	1:1,000	Thermo MA5-15256
Ssa	C-terminal 56 a.a.s	Rabbit polyclonal	1:2,000	Craig lab [58]
Ubiquitin	Full-length bovine Ub	Mouse monoclonal	1:500	Santa Cruz 8017
Rabbit	Rabbit IgG	Goat polyclonal	1:10,000 unless specified	Sigma A0545
Mouse	Mouse IgG	Rabbit polyclonal	1:10,000 unless specified	Sigma A9044
