# Supplementary material for: Prion-Associated Toxicity is Rescued by Elimination of Cotranslational Chaperones
Source: PLoS Genet. 2016 Nov 9;12(11):e1006431. doi: 10.1371/journal.pgen.1006431 (PMC5102407; doi:10.1371/journal.pgen.1006431)
Supplement: S1 References — (PDF) [file pgen.1006431.s014.pdf]

S1 References

55. Derkatch IL, Chernoff YO, Kushnirov VV, Inge-Vechtomov SG, Liebman SW. Genesis and variability of [PSI] prion factors in Saccharomyces cerevisiae. Genetics. 1996 Dec;144(4):1375–86.

56. Stein KC, True HL. Extensive Diversity of Prion Strains Is Defined by Differential Chaperone Interactions and Distinct Amyloidogenic Regions. PLoS Genet. 2014 May 8;10(5):e1004337.

57. Hattendorf DA, Lindquist SL. Cooperative kinetics of both Hsp104 ATPase domains and interdomain communication revealed by AAA sensor-1 mutants. EMBO J. 2002 Jan 15;21(1–2):12–21.

58. Horton LE, James P, Craig EA, Hensold JO. The yeast hsp70 homologue Ssa is required for translation and interacts with Sis1 and Pab1 on translating ribosomes. J Biol Chem. 2001 Apr 27;276(17):14426–33.

59. Vilardell J, Warner JR. Ribosomal protein L32 of Saccharomyces cerevisiae influences both the splicing of its own transcript and the processing of rRNA. Mol Cell Biol. 1997 Apr;17(4):1959–65.

60. Tkach JM, Glover JR. Amino Acid Substitutions in the C-terminal AAA+ Module of Hsp104 Prevent Substrate Recognition by Disrupting Oligomerization and Cause High Temperature Inactivation. J Biol Chem. 2004 Aug 20;279(34):35692–701.
